# Identification of potential microbial risk factors associated with fecal indicator exceedances at recreational beaches

**DOI:** 10.1186/s40793-024-00547-8

**Published:** 2024-01-15

**Authors:** Faizan Saleem, Enze Li, Thomas A. Edge, Kevin L. Tran, Herb E. Schellhorn

**Affiliations:** Department of Biology, McMaster University, 1280 Main St W., Hamilton, ON L8S 4L8 Canada

**Keywords:** Metagenomics, Freshwater beaches, Recreational beaches, Microbiome, Beach action values, Fecal indicators

## Abstract

**Background:**

Fecal bacterial densities are proxy indicators of beach water quality, and beach posting decisions are made based on Beach Action Value (BAV) exceedances for a beach. However, these traditional beach monitoring methods do not reflect the full extent of microbial water quality changes associated with BAV exceedances at recreational beaches (including harmful cyanobacteria). This proof of concept study evaluates the potential of metagenomics for comprehensively assessing bacterial community changes associated with BAV exceedances compared to non-exceedances for two urban beaches and their adjacent river water sources.

**Results:**

Compared to non-exceedance samples, BAV exceedance samples exhibited higher alpha diversity (diversity within the sample) that could be further differentiated into separate clusters (Beta-diversity). For Beach A, Cyanobacterial sequences (resolved as *Microcystis* and *Pseudanabaena* at genus level) were significantly more abundant in BAV non-exceedance samples. qPCR validation supported the Cyanobacterial abundance results from metagenomic analysis and also identified saxitoxin genes in 50% of the non-exceedance samples. *Microcystis* sp and saxitoxin gene sequences were more abundant on non-exceedance beach days (when fecal indicator data indicated the beach should be open for water recreational purposes). For BAV exceedance days, Fibrobacteres, *Pseudomonas*, *Acinetobacter,* and *Clostridium* sequences were significantly more abundant (and positively correlated with fecal indicator densities) for Beach A. For Beach B, Spirochaetes (resolved as *Leptospira* on genus level) *Burkholderia* and *Vibrio* sequences were significantly more abundant in BAV exceedance samples. Similar bacterial diversity and abundance trends were observed for river water sources compared to their associated beaches. Antibiotic Resistance Genes (ARGs) were also consistently detected at both beaches. However, we did not observe a significant difference or correlation in ARGs abundance between BAV exceedance and non-exceedance samples.

**Conclusion:**

This study provides a more comprehensive analysis of bacterial community changes associated with BAV exceedances for recreational freshwater beaches. While there were increases in bacterial diversity and some taxa of potential human health concern associated with increased fecal indicator densities and BAV exceedances (e.g. *Pseudomonas*), metagenomics analyses also identified other taxa of potential human health concern (e.g. *Microcystis*) associated with lower fecal indicator densities and BAV non-exceedances days. This study can help develop more targeted beach monitoring strategies and beach-specific risk management approaches.

**Supplementary Information:**

The online version contains supplementary material available at 10.1186/s40793-024-00547-8.

## Introduction:

Recreational water ecosystems, such as freshwater beaches, are subject to fecal contamination, resulting in beach postings deeming beaches unsuitable for public recreational activities [1, 2]. Deterioration of water quality can cause gastrointestinal illnesses among beachgoers and may be caused by fecal contamination sources, including wastewater treatment plants, septic tank systems, combined sewer overflows and animal/bird feces [3, 4]. Fecal indicator bacteria, including *E. coli* and *Enterococcus,* are correlated with gastrointestinal illness and are thus proxy indicators of water quality for recreational water ecosystems [2, 5]. Despite their common use, fecal indicator bacteria (FIB) are limited by a lack of host specificity and sensitivity [6, 7] and may not necessarily be correlated with the broad spectrum of enteric and non-enteric pathogens of health concern [8]. Additionally, FIB density can be biased due to environmental and physicochemical factors, including water temperature [9], the persistence of FIB for an extended period of time outside the host cell [10], and potential to regrow in beach sediments [11].

Traditional methods for fecal indicator testing in recreational water ecosystems, such as beaches, include culture-based enumeration [12]. *E. coli* and *Enterococcus* are commonly used fecal indicators for beach quality monitoring, and beaches are posted/closed for public visits based on exceedance above Beach Action Value (BAV) thresholds recommended by the United States Environmental Protection Agency [13] and Health Canada [2]. These Beach Action Values are determined based on epidemiological studies (mainly from US Beaches) on the correlation between gastrointestinal diseases among beachgoers/swimmers and fecal indicator densities [1, 14, 15]. However, the occurrence and abundance of fecal indicator bacteria can be variable from different fecal pollution sources [16–18], and may not be indicative of many pathogens of human health concern from non-fecal sources (e.g. toxigenic cyanobacteria).

Molecular methods such as quantitative PCR and digital PCR have been tested as alternatives to augment culturing-based methods for monitoring beach water quality [19, 20]. However, these PCR methods typically rely on a limited number of microbial markers such as *Enterococcus* for FIB monitoring and HF183 for human fecal source tracking, which may not provide a comprehensive overview of the microbial communities in recreational waters. Additionally, obtaining broad microbial information from PCR-based methods requires multiple assays, which can be laborious and costly for complex microbial environments such as water ecosystems.In comparison, a DNA sequencing-based metagenomic approach can simultaneously characterize most of the taxa in a beach water sample and provide gene profiles of the identified organisms [21, 22]. Therefore, a broader range of information obtained from the metagenomics-based approach can potentially provide a valuable screen of microbial changes and improve beach monitoring and management strategies.

The current study evaluates the potential of a metagenomics-based approach to augment beach monitoring strategies by obtaining a comprehensive overview of bacterial community changes associated with Beach Action Value exceedances for recreational freshwater beaches. The questions we focused on are: (1) Do the beach water microbial communities differ significantly between Beach Action Value Exceedance and Non-Exceedance Beach Days? (2) Do the two different beaches show similar patterns of bacterial community changes in response to fecal indicator exceedances? (3) Can the adjacent river and creek water sources account for bacterial changes observed at the beaches? (4) Are there any bacterial taxa of human health concern that do not correlate with fecal indicator densities? (5) Do Antibiotic Resistance Genes (ARGs) abundance correlate with changes in fecal indicator densities?

## Materials and methods

### Study design

**Fig. 1:**
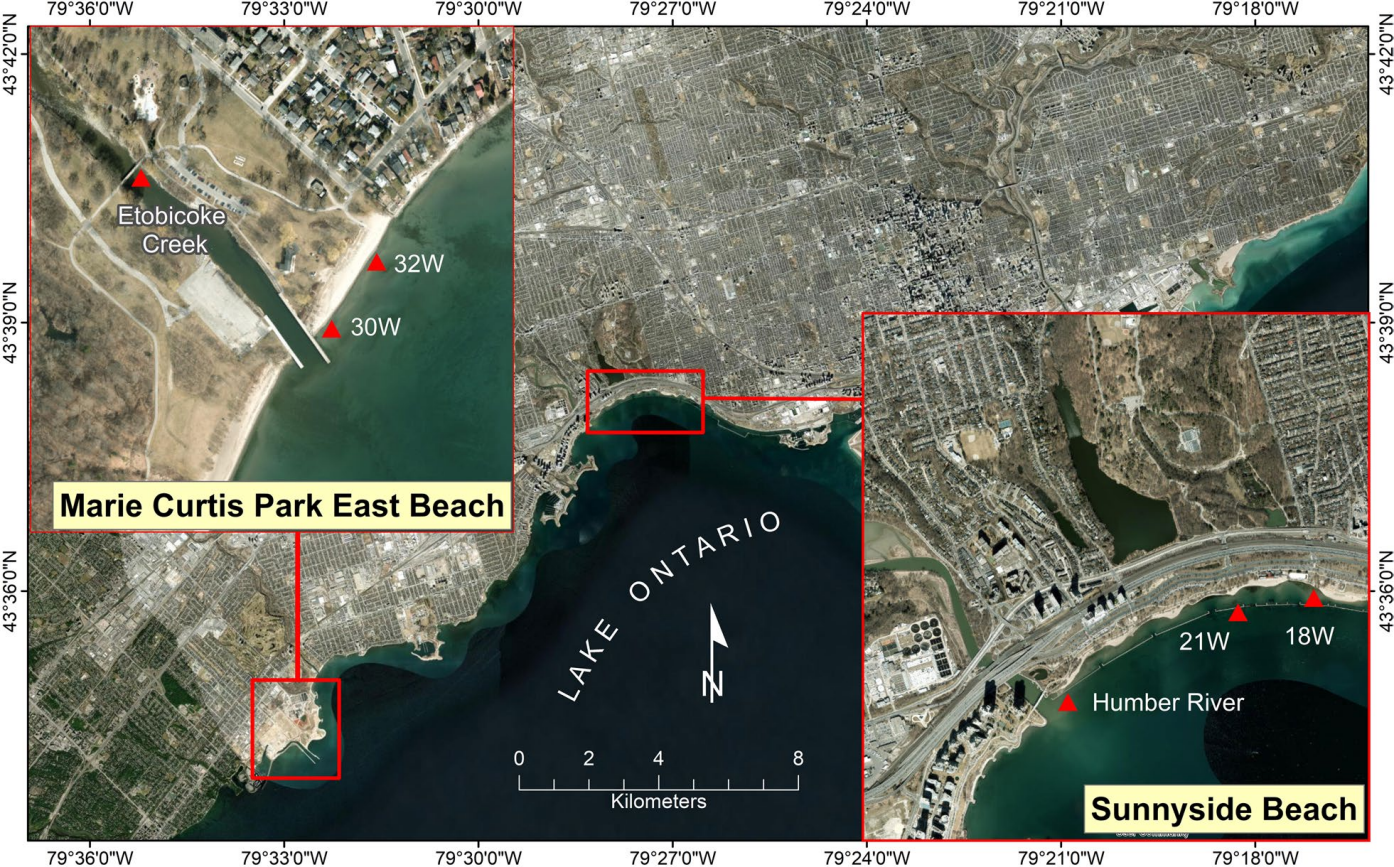
Sampling sites for Marie Curtis Park East Beach, Etobicoke Creek, Sunnyside Beach and Humber River

This study focused on microbial changes associated with fecal contamination at two Toronto recreational freshwater beaches, including Marie Curtis Park East Beach (referred to as Beach A) and Sunnyside Beach (referred to as Beach B), along with their adjacent river and creek water sources as assessed by shotgun DNA sequencing. The water sampling was performed 3 days a week for the summer season 2021 (June 1–August 26). On each sampling day, eight samples were collected, including one each from Etobicoke Creek (referred to as River A and source water for MCPEB) and Humber River (referred to as River B and source water for Sunnyside Beach), and three from each beach transect 30W (43.585610–79.540054), 30W replicate, and 32W (43.585110–79.540560) for Marie Curtis Park East Beach, and transect 18W (43.636612–79.452670), 21W (43.637110–79.457530) and 21W replicate for Sunnyside Beach. These sampling sites and the names used (30W, 32W, 18W, and 21W) were selected according to Toronto Public Health's Beach Monitoring Program (Fig. 1). A total of 309 samples corresponding to 38 beach days were collected. The samples were collected between 5:30 and 7 am and were transported (on ice) to the lab within 1 h for further processing.

### Water sample collection, filtration and DNA extraction

Water samples were collected 30 cm below the water surface in sterile screw-capped polyethylene terephthalate (PET) bottles (1000 mL). For *E. coli* culturing and *Enterococcus* qPCR, a 100 mL sample was passed through a 0.45 µm mixed cellulose esters (MCE) filter (Millipore Corp., Bedford, MA), while for metagenomic DNA extraction, 100 mL of water sample was filtered through 0.22 µm nitrocellulose filters (Millipore Corp., Bedford, MA). For *Enterococcus* qPCR, we used the DNA extraction protocol as described previously [23]. Briefly, the membrane filters were placed in a 2 mL microcentrifuge tube with 0.3 gm glass beads (600 µL of 0.2 µg/mL Salmon sperm DNA as extraction buffer) and bead beaten for 60 s, followed by centrifuging for 60 s at 12, 000 g. The supernatant (400 µL) was collected in a 1.5 mL tube and centrifuged for 5 min at 12, 000 g to collect DNA extract (~ 350 µL) for qPCR. For DNA Sequencing, DNA extraction was performed by using the Norgen Soil Plus DNA Extraction kit (Norgen Biotek Corp., Canada) as described previously [24], followed by DNA quantification using Qubit fluorometer (dsDNA High-Sensitivity Assay kit, Thermo Fisher Scientific, USA).

### *E. coli* enumeration by culturing

*E. coli* enumeration was performed for all samples using Differential Coliform Agar (OxoidTM) with cefsulodin as described previously [23]. Beach water samples were directly processed (100 mL), while Creek/River samples were diluted 1:10 for *E. coli* culturing. The membrane filters were placed on 47 mm agar plates and incubated for 24 h at 44.5 °C [25]. The phosphate buffer saline (passed through the membrane filters) was used alongside the water sample as a negative control. Each sample was tested in triplicate, and enumeration counts for each sampling site were recorded as the mean.

### Enterococcus qPCR

*Enterococcus* qPCR was performed as described previously [23, 26] using Method 1609.1: *Enterococci in Water by TaqMan Quantitative Polymerase Chain Reaction (qPCR) with Internal Amplification Control (IAC) Assay* [27]. In brief, the stock cultures (10^9^ CFUs) of *Enterococcus faecalis* (ATCC 2921) were used to prepare calibrator-positive controls (10^4^ CFUs) and for DNA extraction to prepare Standard Curves. For standard curves, DNA was extracted from the stock cultures using the Norgen Soil Plus Extraction kit described in the previous section. The DNA quantification was done using a Qubit fluorometer (dsDNA High-Sensitivity Assay kit, Thermo Fisher Scientific, USA), followed by the calculation of Target Sequence Copies. Ten-fold dilutions (10–40,000 Target Sequence Copies) were prepared from Stock Culture DNA, and four individual standard curves were used to create a composite standard curve. Alongside each batch of samples, two calibrator positive controls, two method blanks (phosphate buffer saline passed through filters), and two non-template controls (DNA extract replaced by nuclease-free water) were analyzed to test for DNA extraction efficiency and contamination. Each PCR reaction was carried out in duplicate and comprised DNA Recovery Control (Salmon DNA qPCR) and a PCR inhibition control (Internal Amplification Control). Each qPCR reaction (25 µL) comprised of 12.5 µL TaqMan Environmental Master mix (Thermo Fisher Scientific, USA), 3.0 µL of primer–probe working solution (primers and probes concentration was 1.0 µM and 80.0 nm, respectively), 2.0 µL of internal amplification control and 2.5 µL Bovine Serum Albumin (2 mg/mL). All the reactions were performed on Bio-Rad CFX96 Touch Real-Time PCR (Bio-Rad Inc. USA). The *Enterococcus* Calibrator Cell Equivalents were calculated using an Excel sheet (https://www.epa.gov/cwa-methods/other-clean-water-act-test-methods-microbiological#file-183743) provided by USEPA.

### Sample selection criteria for shotgun DNA sequencing

The following considerations were used for sample selection: (1) samples having *E. coli* > 235 CFU/100 mL by plate counting or having *Enterococcus* > 1000 calibrator cell equivalents by qPCR as determined previously [23, 26] were defined as BAV exceedances, (2) Sampling Days showing exceedance or non-exceedance for all the sampling sites for a beach were prioritized, (3) an equal number of beach samples were selected for BAV Exceedance and Non-exceedance groups, (4) for a single Beach Day, DNA from all the sampling sites for a beach was pooled and, (5) For creek/river water sources (Etobicoke Creek and Humber River), sampling dates matching to the selected beach samples were sequenced for comparison of bacterial trends with associated beaches. In total, 48 pooled water samples were selected for shotgun sequencing (Additional file 1: Table 1).

### Shotgun DNA sequencing and quality control analysis

To avoid DNA concentration bias during library pooling, input DNA from each sample was normalized to 200 ng. The library was prepared using NEBNext® Ultra™ II DNA library preparation kit with TruSeq3 paired-end adapters. The fragment size and read length were 500 bp and 150 bp, respectively. DNA sequencing was performed on Illumina NextSeq 2000 (2 × 150) at the Farncombe Sequencing Institute at McMaster University. The quality of raw reads was analyzed using FASTQC [28]. Adapter trimming, decontamination (removal of reads mapping to human), quality filtration (Quality Score > 30, by the sliding window algorithm, widowsize = 4 bases), Length Filtration (> 75 bp) and removal of tandem repeats were performed using the KneadData pipeline (Available at: http://huttenhower.sph.harvard.edu/kneaddata).

### Cyanobacteria and cyanotoxin gene qPCR

The number of Cyanobacterial and Cyanotoxin (Microcystin, Saxitoxin and Cylindrospermopsin) gene copies from metagenomics sequencing were validated using CyanoDTec Total Cyanobacteria and Toxin Kit (Phytoxigene™) according to the manufacturer's instructions. In total, 17 (8 for non-exceedance and 9 for exceedance beach days) pooled DNA samples from Marie Curtis Park East Beach were used for Cyanobacteria/toxin qPCR. Four standard curves were run separately for each assay (Total Cyanobacteria and Toxin), followed by preparing a composite standard curve. The standard curve range for each assay was 10–100,000 gene copies. Each reaction comprised 20 µL of mastermix/primer–probe solution and 5 µL of DNA from pooled samples (the same DNA used for shotgun sequencing). The gene copy numbers for each sample were calculated using the Slope-Intercept equation from the composite standard curve and normalized to gene copies per nanogram of DNA. A gene was only considered present in a sample if the gene copies/Threshold-cycle values were within the range of the standard curve, and the results were only accepted if the internal amplification control threshold-cycle (Ct) value for a sample was not offset more than 1.5 compared to non-template control.

### Bioinformatics and data analysis

Clean reads were aligned against NCBI RefSeq protein Database (Accessed on February 24 2023) using DIAMOND BLASTx on sensitive mode [29], followed by annotation using MEGAN6 (Weighted Lowest Common Ancestor (LCA) method: Minimum-Score = 50, Top-percent filter = 10%, Minimum-Support = 50) [30, 31]. For lateral comparison, annotated reads were rarefied (normalized) to the sample of the smallest size (~ 4.8 million reads) to neutralize bias associated with sequencing depth [31–33]. For the Core Microbiome Analysis, bacterial genera present in ≥ 50% of the samples with a relative abundance of ≥ 0.1% were selected [34, 35]. For the Alpha diversity analysis, Shannon–Weaver and Simpson's Reciprocal diversity indices measurements were calculated [36, 37]. Two alpha diversity matrices were used for cross-validation of in-sample diversity. For Antibiotic resistance genes (ARGS) analysis, quality-filtered sequences were assembled into contigs using the MEGAHIT (Metagenome Assembler: k_min + 1 = 2, Min = kmer Size = 21, Max kmer Size = 99, k-step = 20, and minimum-contig size = 200) [38], followed by ARGs annotation using Pathofact pipeline (Combines DeepARG and Resistance gene identifier results for cross-validation) [39]. Statistical analysis was performed using STAMP metagenomic data statistical analysis software [40]. Analysis of Similarity (ANOSIM) was used for the Beta-Diversity assessment [24]. Shapiro–Wilk's normality testing (Stats v3.6.2 R package) was used to determine the normal distribution of the tested microbial variables, followed by either Welch's (and one-way ANOVA) or Wilcoxon-Mann–Whitney t-test for comparison between the groups [41] and Spearman's rank test (Log-transformed data) for correlation analysis between the variables [42, 43].

## Results

### Quality control analytics

A total of 48 pooled DNA samples from recreational beaches and associated creek/river water were sequenced (Additional file 1: Table 1). Next-generation DNA sequencing provided 12.9 ± 3.2 and 13.7 ± 3.5 million raw reads for Beach A and B, respectively (Additional file 1: Table 2). A high proportion (83%) of raw reads passed the quality control criteria and were processed for downstream taxonomic/functional analysis. The R^2^ value for the qPCR composite standard curves (*Enterococcus*, Total Cyanobacteria and Toxin Assays) was within 0.992–0.999, while slope and intercept values were between − 3.25 to − 3.46 and 38.66 to 39.13, respectively (Additional file 1: Table 2). Additional file 1: Figure 2 shows the taxonomic identification after annotation/Least Common Ancestor (LCA) analysis. Bacterial sequences dominated all the samples (93–96%), followed by eukaryotes (3–4%), viruses (1–2%) and archaea (< 1%). The results below highlight bacterial diversity/composition changes associated with the Beach Action Value exceedance and non-exceedance of fecal indicator bacteria.

### Diversity between beach action value (BAV) exceedance and non-exceedance beach days

**Fig. 2:**
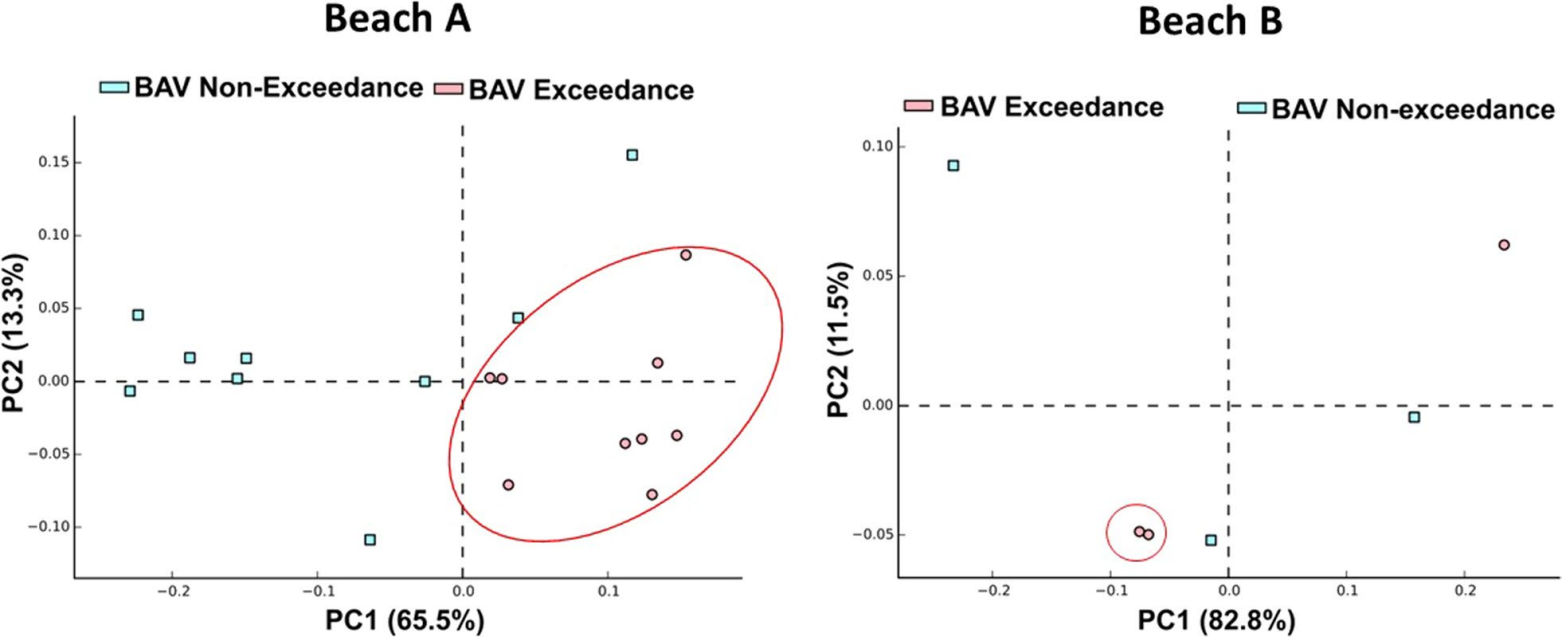
Differential abundance principal component analysis plots for beach action value (BAV) exceedance and non-exceedance samples from Beach A and Beach B

Alpha diversity (diversity within the samples) was approximately 10% higher in Beach Action Value (BAV) Exceedance day samples than the non-exceedance samples for both Beach A (4.4 ± 0.5 versus 4.0 ± 0.3) and Beach B (4.1 ± 0.1 versus 3.7 ± 0.7) (Table 1). Interestingly, exceedance samples from Beach A showed a ~ 9% higher alpha diversity than associated river source. Additionally, when concatenated at the genus level, exceedance samples were segregated into separate clusters from non-exceedance samples for both beaches on principal component analysis plots (Fig. 2 and Additional file 1: Figure 3). Analysis of core microbiome differences identified 9 (24%) and 3 (10%) bacterial genera exclusive to BAV Exceedance beach days from Beach A and Beach B, respectively (Additional file 1: Figure 4).

**Table 1:** Alpha diversity analysis for beach water samples from Beach A, Beach B and associated river sources

Microbial diversity within the samples (alpha diversity)	Beach action value exceedance day (mean ± standard deviation)	Beach action value non-exceedance day (mean ± standard deviation)	Sampling site
Shannon–weaver diversity matrix	4.4 ± 0.5	4.0 ± 0.3	Beach A
	4.1 ± 0.1	3.7 ± 0.7	Beach B
Simpson-reciprocal diversity matrix	8.8 ± 1.9	8.4 ± 1.8	Beach A
	8.3 ± 1	7.9 ± 3.7	Beach B

	Creek/river samples (mean ± standard deviation)		
Shannon–weaver diversity matrix	4.0 ± 0.8		River A
	4.1 ± 0.6		River B
Simpson-reciprocal diversity matrix	7.8 ± 3.4		River A
	7.5 ± 3.2		River B

### Bacterial community changes associated with fecal indicator exceedances/non-exceedances

**Fig. 3:**
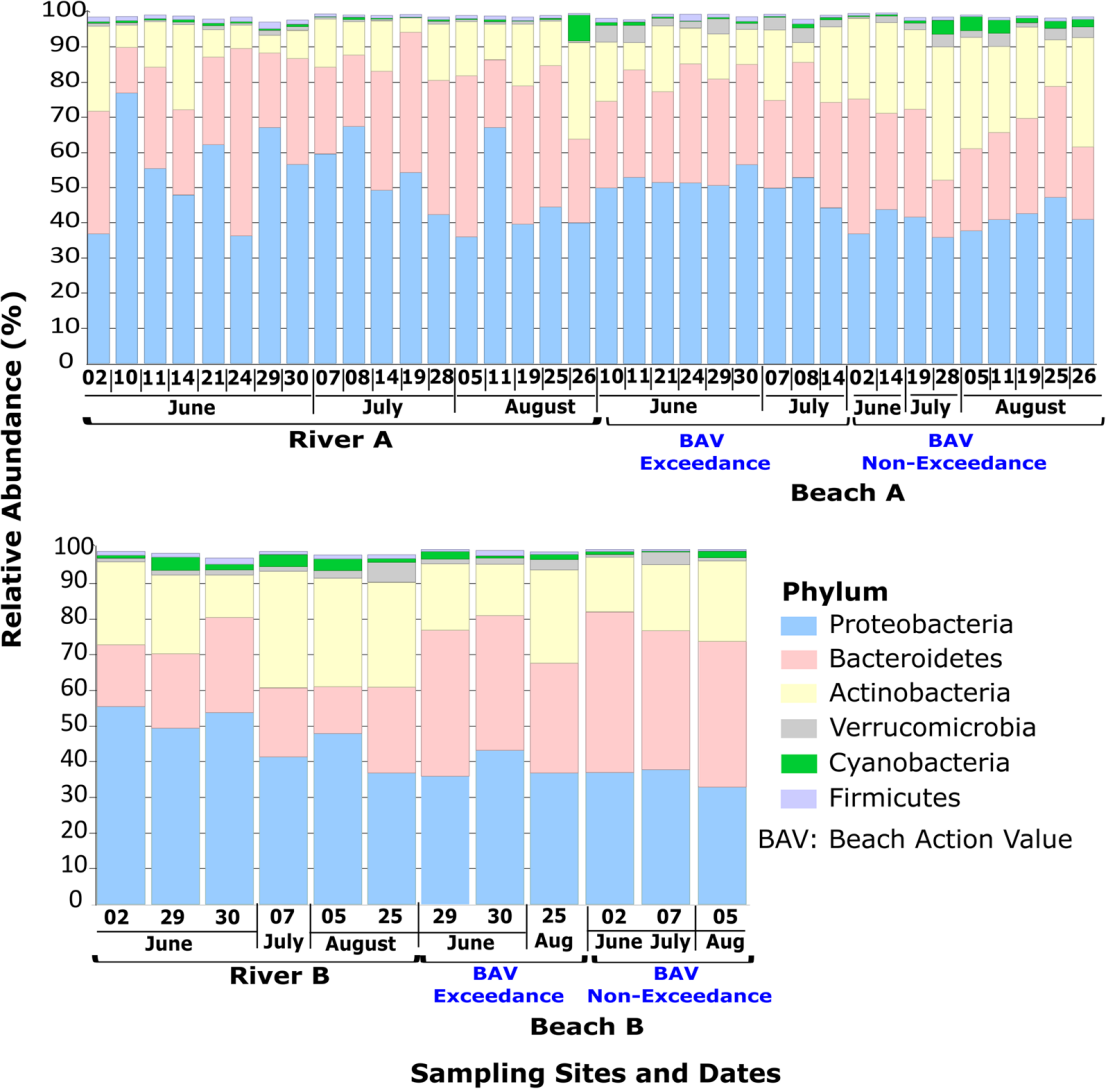
Relative abundance profile for six top most abundant phyla identified in beach action value exceedance and non-exceedance beach day samples

**Fig. 4:**
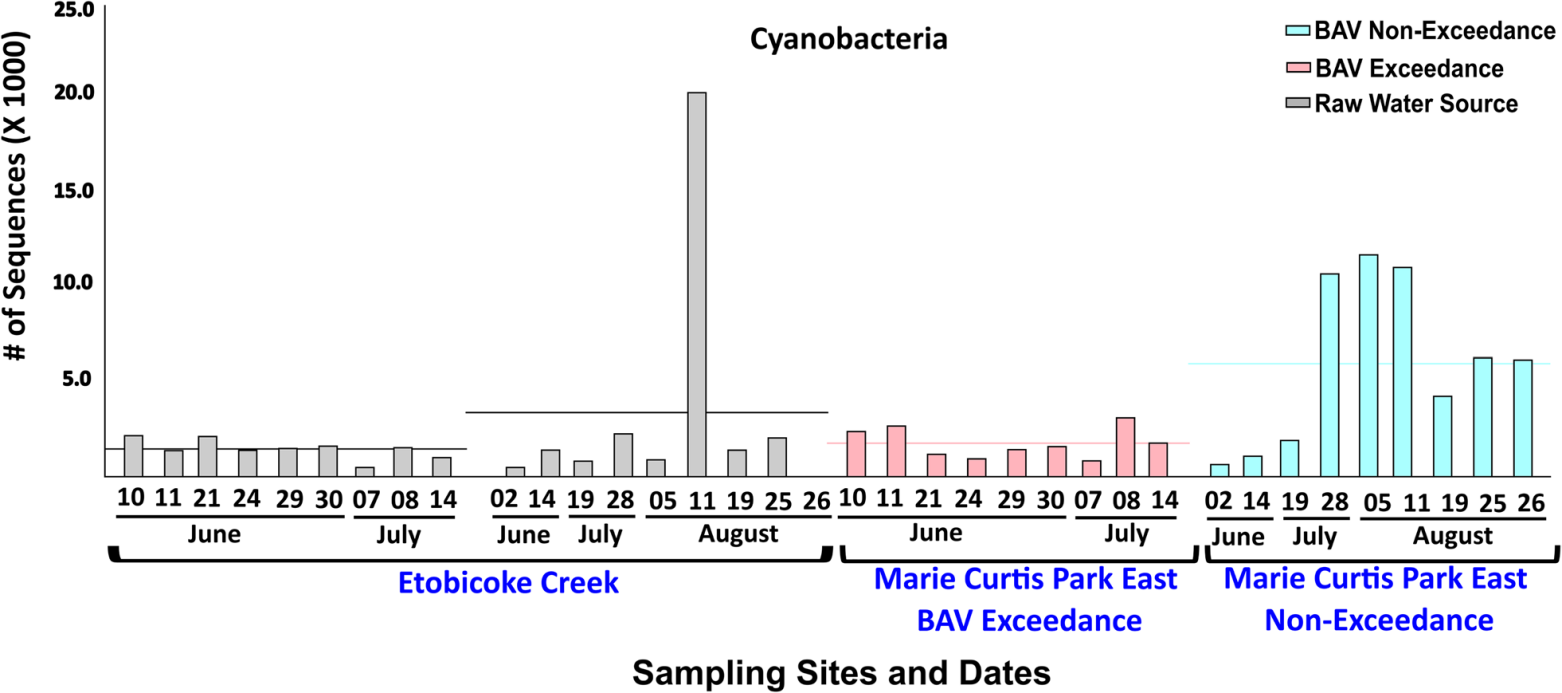
Bar plot of cyanobacterial abundance for samples from Marie Curtis Park East Beach and Etobicoke Creek. The horizontal line represents the average number of normalized sequences for each group

Bacterial community composition on the phylum level was similar for both beaches (Fig. 3). Proteobacteria (38–75%) were the most abundant, followed by Bacteroidetes (20–40%), Actinobacteria (10–30%), Verrucomicrobia (5–7%), Cyanobacteria (2–4%) and Firmicutes (1–2%). For Beach A, at the phylum level, Proteobacteria (*p* = 1.04e−5) and Fibrobacteres (*p* = 0.049) were significantly more abundant on Beach Action Value Exceedance days, while Actinobacteria (*p* = 7.64e−4) and Cyanobacteria (*p* = 0.021) were significantly more abundant on non-exceedance beach days (Additional file 1: Figure 5). Compared to corresponding sampling dates from beach and river, Cyanobacterial sequences were more abundant for non-exceedance beach samples (Fig. 4). Fibrobacteres sequences were more abundant for exceedance samples and showed a similar abundance pattern between river A and beach A samples (Additional file 1: Figure 6). Additional file 1: Figures 7 and 8 show that Cyanobacterial sequences classified into *Microcystis* and *Pseudanabaena* genera. Metagenomics analyses indicated that *Microcystis* sequences were absent in exceedance samples but detected in 6/9 = 67% of non-exceedance samples (and 100% of samples from late July through August). *Pseudanabaena* sequences were detected in both exceedance and non-exceedance samples. However, for *Pseudanabaena*, we did not observe any statistically significant (*p* = 0.36) difference between BAV exceedance and non-exceedance samples.

**Fig. 5:**
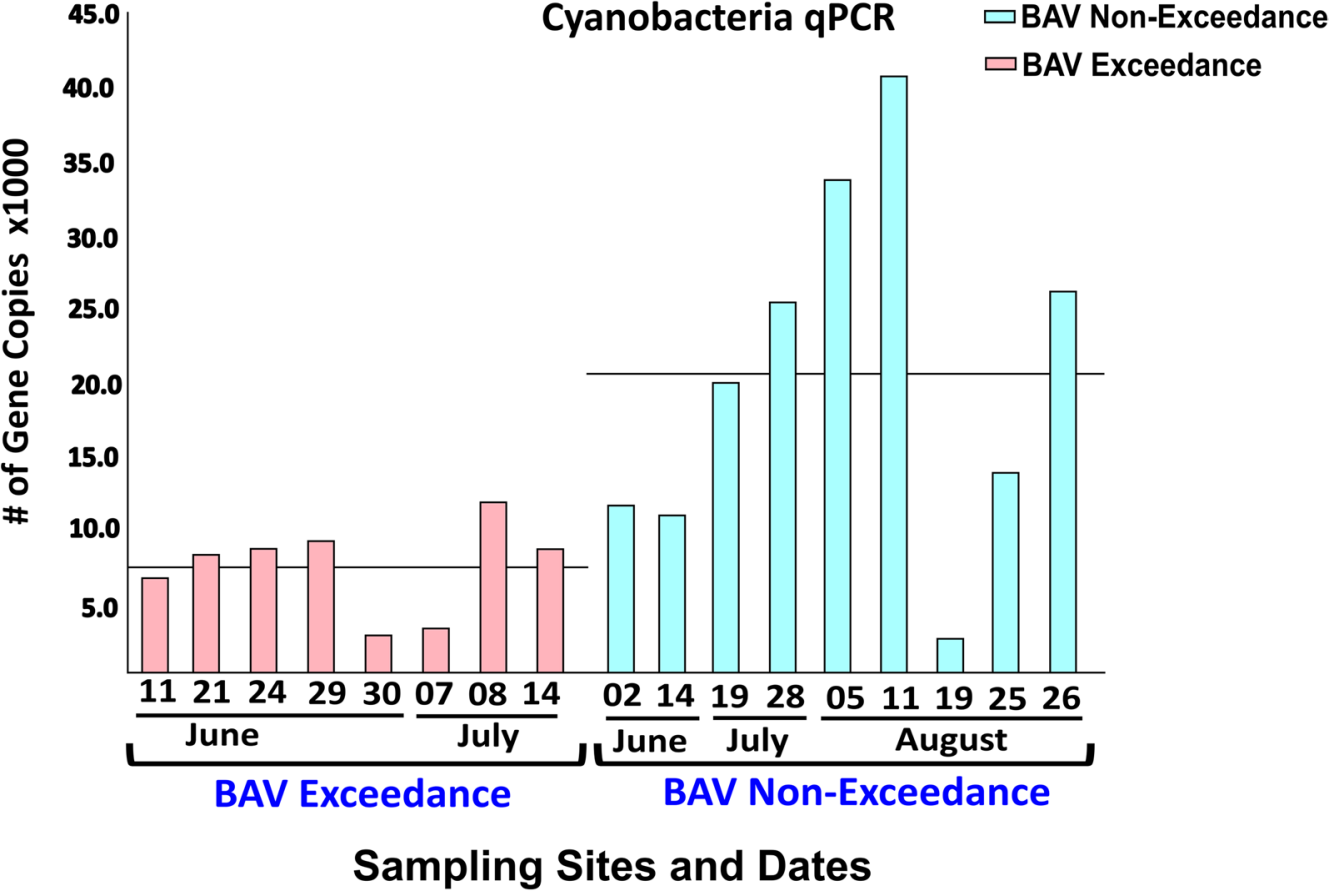
Bar plot of *Microcystis* abundance measured by qPCR for Beach A samples. The horizontal line represents the average number of gene copies for each group

We further validated the Cyanobacterial findings using 16S rRNA (Cyanobacterial-specific) qPCR (Fig. 5). Cyanobacterial-specific qPCR results confirmed the metagenomic analysis. qPCR results validated that BAV-non-exceedance beach days had significantly higher (*p* = 9.99e−4) cyanobacterial gene copies compared to BAV exceedance days for Beach A. Although qPCR did not detect Cyanobacteria-associated microcystin and cylindrospermopsin genes (Table 2), 50% (4/8) of the qPCR tested BAV non-exceedance samples (corresponding to samples with higher Cyanobacterial gene copies) showed the presence of saxitoxin genes.

**Table 2:** Cyanotoxin presence/absence for BAV exceedance and Non-exceedance samples from Marie Curtis Park East Beach

Date	Beach action value status	Microcystin/nodularin	Cylindrospermopsin	Saxitoxin	Saxitoxin gene copies
June 11	Exceedance	−	−	−	−
June 21		−	−	−	−
June 24		−	−	−	−
June 29		−	−	−	−
June 30		−	−	−	−
July 7		−	−	−	−
July 8		−	−	−	−
July 14		−	−	+	2284
June 2	Non-exceedance	−	−	−	−
June 14		−	−	−	−
July 19		−	−	+	76
July 28		−	−	+	145
August 5		−	−	+	12,079
August 11		−	−	+	400
August 19		−	−	−	−
August 25		−	−	−	−
August 26		−	−	−	−

For Beach B, at the phylum level, only Spirochaetes sequences were significantly (*p* = 0.016) more abundant in BAV exceedance samples than non-exceedance samples, and corresponding sampling dates from River B also showed higher mean abundance for sampling dates corresponding to BAV exceedance beach samples (Additional file 1: Figure 9). At the genus level, Spirochaetes sequences were mainly classified into *Leptospira* and showed comparatively higher mean abundance in BAV exceedance samples (Additional file 1: Figure 10), though the difference was not significant (*p* = 0.25).

**Fig. 6:**
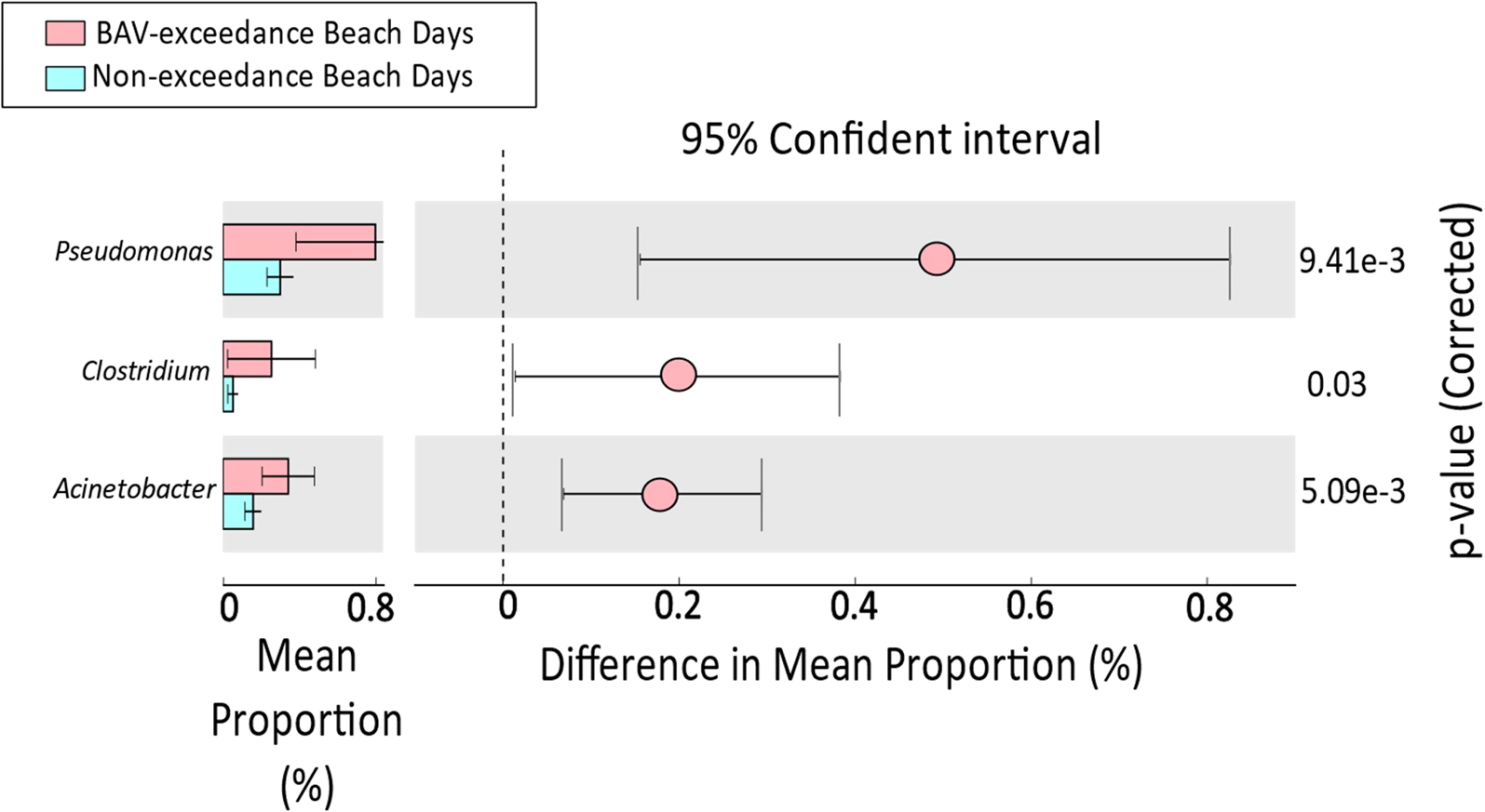
Differential abundance of statistically significant bacterial genera between beach action value exceedance and non-exceedance beach days from Beach A

For Beach A, at the genus level, abundances of *Pseudomonas* (*p* = 9.41e−3), *Acinetobacter* (*p* = 5.09e−3), and *Clostridium* (*p* = 0.038) were significantly higher for BAV exceedance days than non-exceedance day samples (Fig. 6). Compared with non-exceedance beach samples, the abundances of *Pseudomonas* and *Acinetobacter* were higher in both Etobicoke Creek and Marie Curtis Park East Beach for the sampling dates corresponding to Beach Action Value Exceedances (Additional file 1: Figures 11 and 12). Table 3 shows the correlation between fecal indicator densities and differentially abundant genera for Beach A and River A. For the Beach A samples, the fecal indicator densities (*E. coli* by culture and *Enterococcus* by qPCR) showed a significant positive correlation with *Pseudomonas* (r_s_ = 0.7, p < 0.01), *Acinetobacter* (r_s_ = 0.6 and 0.5, p ≤ 0.01), and *Clostridium* (r_s_ = 0.5, p ≤ 0.05). River A samples also showed a significant positive correlation between fecal indicator densities with *Pseudomonas* (r_s_ = 0.7 and 0.8, p < 0.01) and *Acinetobacter* (r_s_ = 0.5, *p* = 0.01).

**Table 3:** Correlation analysis between fecal indicator densities and differentially abundant bacterial genera for Beach A and River A

Fecal indicator	Bacterial genera	Correlation coefficient (r_s_)	*P* value	Sampling site
*E. coli*	*Vibrio*	0.1	0.5	Beach A
	*Clostridium*	0.5	0.03	
	*Acinetobacter*	0.6	0.002	
	*Pseudomonas*	0.7	5e−4	
*Enterococcus*	*Vibrio*	0.2	0.3	Beach A
	*Clostridium*	0.6	0.008	
	*Acinetobacter*	0.5	0.01	
	*Pseudomonas*	0.7	1e−4	
*E. coli*	*Vibrio*	0.3	0.1	River A
	*Clostridium*	0.3	0.1	
	*Acinetobacter*	0.5	0.01	
	*Pseudomonas*	0.7	5e−4	
*Enterococcus*	*Vibrio*	0.5	0.03	River A
	*Clostridium*	0.3	0.1	
	*Acinetobacter*	0.5	0.01	
	*Pseudomonas*	0.8	2.4e−5	

**Fig. 7:**
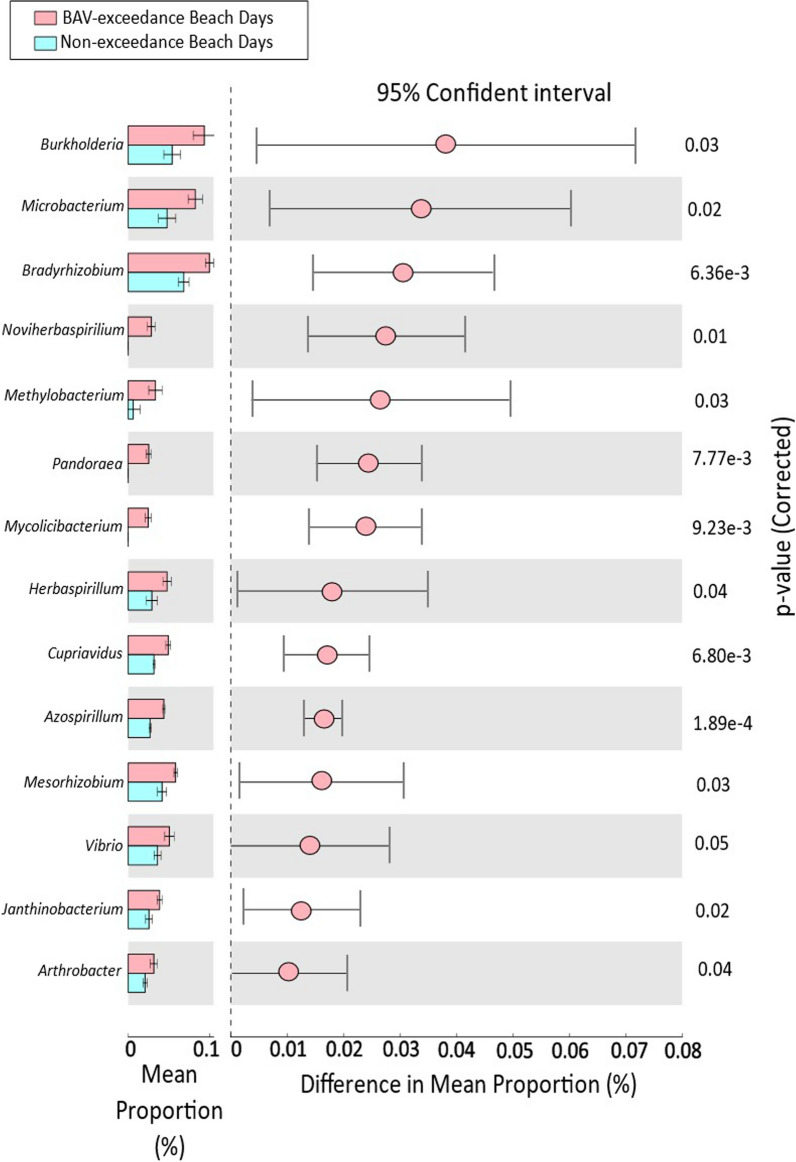
Differential abundance of statistically significant bacterial genera between beach action value exceedance and non-exceedance beach days from Beach B

For Beach B, at the genus level, 14 genera, including *Burkholderia* (*p* = 0.034) and *Vibrio* (*p* = 0.05), were significantly more abundant on BAV exceedance days than non-exceedance day samples (Fig. 7). The mean abundance of *Burkholderia* and *Vibrio* was also higher in River B on the sampling dates corresponding to BAV exceedance at Sunnyside Beach (Additional file 1: Figures 13 and 14). For correlation with fecal indicator densities, only the *Burkholderia* counts showed a significant positive correlation with *E. coli* density for Beach A (Table 4).

**Table 4:** Correlation analysis between fecal indicator densities and differentially abundant bacterial genera for Beach B and River B

Fecal indicator	Bacterial genera	Correlation coefficient	*P* value	Sampling site
*E. coli*	*Leptospira*	0.2	0.7	Beach B
	*Burkholderia*	0.8	0.05	
	*Vibrio*	0.7	0.1	
	*Clostridium*	0.4	0.3	
*Enterococcus*	*Leptospira*	0.3	0.4	Beach B
	*Burkholderia*	0.4	0.3	
	*Vibrio*	0.6	0.2	
	*Clostridium*	0.4	0.4	
*E. coli*	*Leptospira*	0.7	0.1	River B
	*Burkholderia*	0.5	0.2	
	*Vibrio*	0.4	0.3	
	*Clostridium*	0.8	0.03	
*Enterococcus*	*Leptospira*	0.5	0.2	River B
	*Burkholderia*	0.6	0.1	
	*Vibrio*	0.02	1	
	*Clostridium*	0.4	0.3	

*Escherichia* sequences were detected in more samples than *Enterococcus* (Additional file 1: Figures 15 and 16). *E. coli* sequences were detected in 90% of the samples for both beaches, while *Enterococcus* sequences were detected in only one sample for each beach. Additionally, the difference in mean proportions between BAV exceedance and non-exceedance samples for *Escherichia* was not significant (p > 0.05) for both beaches.

### Relationship between antibiotics resistance genes and fecal indicator exceedances/non-exceedances

**Fig. 8:**
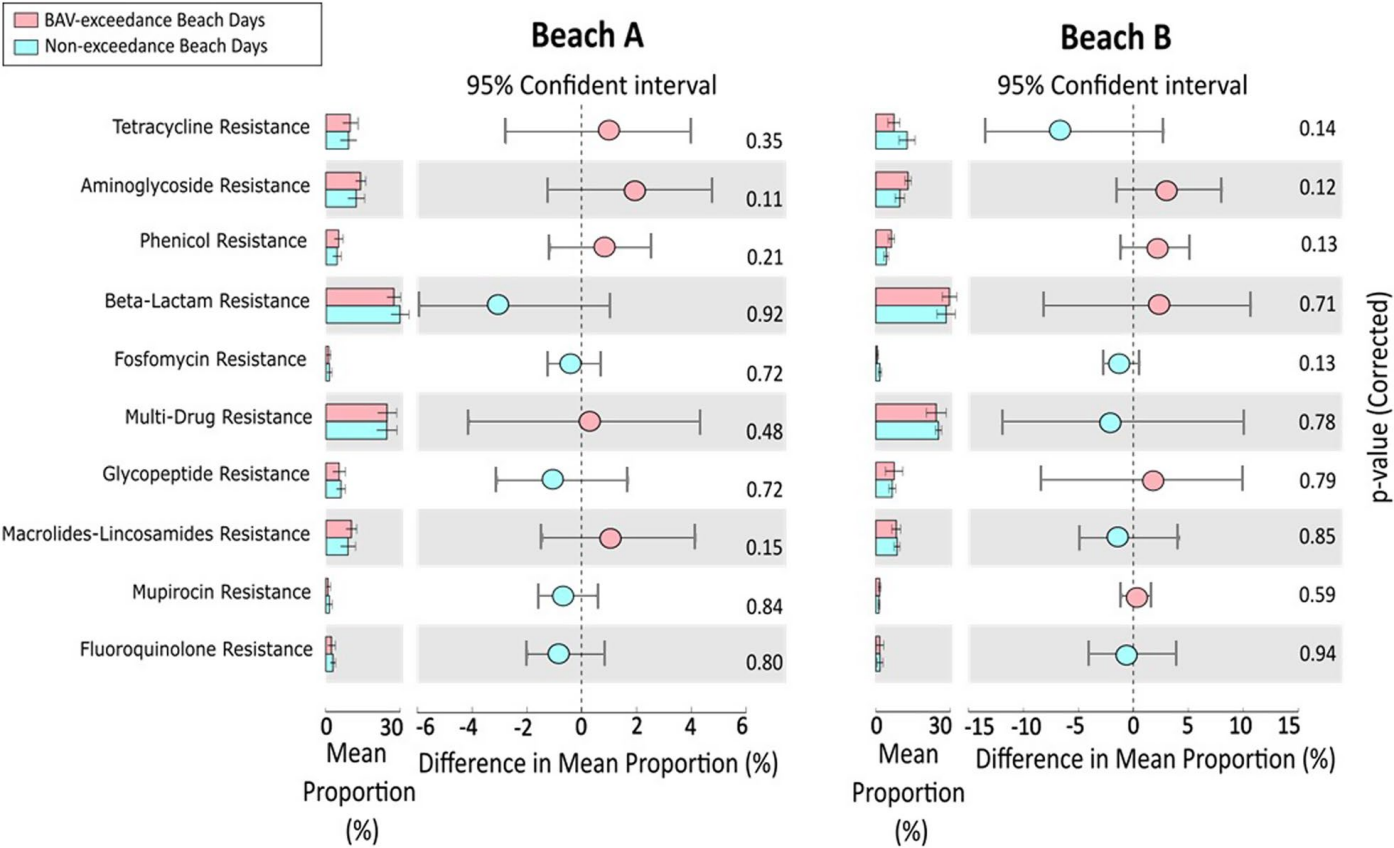
Differential abundance of antibiotic resistance genes in beach action value exceedance and non-exceedance beach days from Beach A and Beach B

Antibiotic resistance analysis revealed that for both Marie Curtis Park East and Sunnyside Beaches, the five most abundant resistant gene groups were those involved in Beta-lactam antibiotic resistance, Multidrug resistance efflux pumps, Aminoglycoside resistance, Macrolides-Lincosamides resistance and Tetracycline resistance (Fig. 8). However, the difference in mean proportions for the antibiotic-resistance genes was not statistically significant (p > 0.05) between the Beach Action Value Exceedance and Non-exceedance days for both beaches. Additionally, there was no significant correlation between fecal indicator densities and antibiotic resistance gene abundances (Additional file 1: Table 3).

## Discussion

Freshwater Beach monitoring programs use densities of fecal indicators, including *E. coli* and *Enterococcus,* as a reference for evaluating beach water quality. For each fecal indicator and analysis method (enumeration by culturing or quantitative PCR), a specific Beach Action Value (BAV) is recommended by the Public Health Authorities, and beaches are posted for recreational uses if the levels of fecal indicator densities are above the BAV [2, 5]. However, limited experimental data is available for microbial community changes associated with Beach Action Value exceedances, and a single fecal indicator may not be indicative of diverse potential health risks from enteric and non-enteric pathogens, toxigenic cyanobacteria and aspects such as antimicrobial resistance (Ferguson et al., 2012; Li et al., 2021). Compared to conventional analysis methods (culturing and PCR), a metagenomics-based approach provides a robust and comprehensive taxonomic and functional screening profile for water ecosystems. Additionally, metagenomic analysis can provide a foundation for targetted water quality monitoring by identifying region/site-specific microbial/functional differences [37, 44]. This study aimed to provide a comprehensive profile of bacterial community changes associated with fecal indicator Beach Action Value Exceedances and Non-exceedances across two urban recreational freshwater beaches.

Alpha diversity for water samples from Great Lakes beaches has been found to range from 3.5 [45, 46] to 7 [47], and our results for Marie Curtis Park East and Sunnyside Beaches were within this range. Beach Action Value Exceedance samples from both beaches showed comparatively higher alpha diversity than non-exceedance and associated creek/river water sources, indicating other bacterial groups may be present in exceedance day samples. Aside from creeks or rivers, sand/sediment resuspension and other fecal pollution sources can impact bacterial diversity in recreational waters, including birds and mammals defecating nearby around the beach ecosystem [3, 48, 49]. Beach Action Value Exceedance and non-exceedance samples from both beaches separated into independent clusters on principle component analysis plots, which indicates bacterial abundance and diversity differences. Bacterial genera that differed between Beach Action Value Exceedance days and Non-exceedance days differed between the two beaches, suggesting localized influences around each beach rather than regional processes drive microbial community changes. Differences in diversity and the core microbiome between BAV exceedance and non-exceedance beach day samples could be due to diverse environmental factors, including rain events that can increase bacterial diversity on BAV exceedance beach days by increasing the bacterial load from urban runoff, creek/river plumes entering lakes, or increased flows dislodging soil and sediment-attached microbial communities [50, 51].

At the phylum level, Proteobacteria, Bacteroidetes, Actinobacteria, Verrucomicrobia, and Firmicutes were abundant in all tested samples, consistent with other findings (surface water and sediment) for the Great Lakes region [24, 52, 53]. Interestingly, for Beach A, Cyanobacteria sequences were significantly more abundant in BAV non-exceedance samples than exceedance days or associated with the adjacent creek water source. Additionally, the increased abundance of Cyanobacteria and Saxitoxin genes was more notable in the later summer weeks (July–August 2021), corresponding to BAV non-exceedance beach days. Thus, we detected *Microcystis*, *Pseudanabaena*, and saxitoxin gene sequences on many non-exceedance beach days when *E. coli* data indicated Beach A should be open for water recreation. Both Beach A and B are not routinely monitored for cyanobacteria or harmful algal species and would typically be tested in response to visual complaints of bloom formations. Cyanobacterial genera, including *Microcystis,* can lead to deteriorating (eutrophication and toxin production) water quality for recreational purposes [54, 55]. Similar to our findings, a study on recreational waters [56] identified decreased fecal indicator densities associated with higher Cyanobacterial (specifically *Microcystis*) levels. Therefore, relying solely on the fecal indicator densities for recreational water quality may provide an incomplete perception of human health risks at beaches. Fibrobacteres species (cellulose-degrading bacteria) are specific to the rumen microbiome of ruminant animals [57], while Spirochaetes are found in farm animals (cows and pigs) but not human fecal material [58]. *Leptospira* contamination can be from domestic and wild animals [59], which may indicate that fecal contamination for Beach B can be from both water sources (Humber River) and localized (wild animals). The increased abundance of Fibrobacteres and Spirochaetes, along with increased *E. coli* levels, on BAV exceedance days at our two beaches may indicate fecal contamination from livestock or other ruminants in runoff to river sources that subsequently impacts the beaches.

On the genus level, *Pseudomonas*, *Acinetobacter* and *Clostridium* were significantly more abundant in BAV exceedance samples from Beach A (and River A samples for the same dates), while *Bulkholderia* and *Vibrio* were more abundant for BAV exceedance samples from Beach B (and River B samples for the same dates). Studies in the Great Lakes region [24, 50] have identified *Pseudomonas, Clostridium* and *Acinetobacter* as common genera in stormwater, which may explain the influx of these genera into Beach A from the adjacent creek water source. Both River A and River B are significantly impacted by stormwater systems at times, contributing to increased fecal contamination at Marie Curtis Park East and Sunnyside Beaches [60, 61]. *Burkholderia* and *Vibrio* sequences have been identified to be associated with both human and animal fecal contamination in urban recreational waters [62], which is in agreement with our findings for Beach B and may indicate fecal contamination from both associated waters (Humber River) and localized (wild animals) sources.

The metagenomics method also provided a screen for detecting a diverse range of antibiotic resistance genes. We detected numerous Antibiotic Resistance Genes (ARGs) of clinical concern in water samples from both Beaches. Similar to our beaches, other studies have also identified ARGs at beaches [63] and across ~ 350 lakes in Canada [64]. We found no significant association between numbers of antibiotic resistance genes (ARGs) and fecal indicators, with exceedances based on *E. coli* or *Enterococcus* numbers, this suggests the occurrence of AMR genes was not solely driven by AMR genes associated with these two FIBs. AMR genes are likely associated with diverse other bacteria, including those unrelated to fecal pollution, which is another limitation of using traditional culture (*E. coli*) or qPCR (*Enterococcus*) methods to predict overall AMR gene occurrence. However, ARG pools were consistently present at our two beaches, with the potential for horizontal gene transfer to bacterial species of human health concern. While water can play a role in routes of ARG exposure, quantifying that role and its associated human health risks requires further research [65].

Overall, this proof of concept study demonstrates the potential value of metagenomics for enabling a more comprehensive screen of bacterial community changes associated with fecal indicator Beach Action Value Exceedance and Non-exceedance conditions at freshwater beaches. The differences in bacterial diversity and abundance in response to BAV exceedances for Beach A were more pronounced (supported by correlation test) compared to Beach B. This may be due to Beach A sampling locations being closer to the mouth of River A than the proximity of Beach B sampling locations to the mouth of River B. In addition, a break wall limits the direct influx from River B into Beach B, while Beach A is on an open coastline that receives unhindered water flow directly from River A under the right wind and current conditions. One limitation of this study is the localization of both tested beaches in the same geographical location or close proximity. However, we identified site-specific microbial differences between the two tested beaches, and future studies can build on our results/methodology to include a larger study area. Additionally, Our results provide insight into localized processes influencing bacterial community changes at freshwater beaches and further identify limitations of existing culture-based and single-gene PCR assay approaches for assessing recreational water quality. The results provide a foundation to guide more comprehensive screening for harmful microorganisms, as well as toxin and antimicrobial resistance genes, in order to improve recreational water quality monitoring and enable more targeted and site-specific risk management strategies.

## Conclusion

1. Cyanobacterial sequences (*Microcystis* and saxitoxin genes in particular) were significantly more abundant in Beach Action Value Non-exceedance samples from Beach A, demonstrating that fecal indicator bacteria densities may not indicate health risks associated with harmful algal blooms and the eutrophication of recreational waters.
2. The increase of Fibrobacteres sequences in BAV exceedance days of Beach A may represent an influx of fecal contamination from livestock or other ruminant animals.
3. *Pseudomonas*, *Acinetobacter,* and *Clostridium* sequences were significantly more abundant on the BAV exceedance days and positively correlated with fecal indicator densities at Beach A.
4. The increase of Spirochaetes (specifically *Leptospira*), *Burkholderia*, and *Vibrio* was significantly associated with Beach Action Value Exceedance samples from Beach B.
5. Similar bacterial diversity and abundance trends between beach and river samples suggest the Creek and River are primary sources of bacterial contamination at the beaches.
6. Pools of Antibiotic Resistance Genes (ARGs) were consistently detected at both beaches, indicating potential for transfer to potentially pathogenic genera by horizontal gene transfer.
7. The metagenomics approach provided the capability of extending beyond *E. coli* and single gene PCR testing to provide a comprehensive screen of beach water samples for bacterial community composition and toxin and antimicrobial resistance genes associated with changing beach water conditions.

### Supplementary Information

**Additional file 1**. Supplementary Tables 1–3 and supplementary Figures 1–16. 

## Data Availability

The sequencing data from this study is available from the corresponding author upon reasonable request. All the analytical data supporting the findings in this study is provided in the figures and tables in the main manuscript and supplementary data files.
