# Supplementary material for: Identification of potential microbial risk factors associated with fecal indicator exceedances at recreational beaches
Source: Environ Microbiome. 2024 Jan 15;19:4. doi: 10.1186/s40793-024-00547-8 (PMC10790499; doi:10.1186/s40793-024-00547-8)
Supplement: Supplementary file 1 — Additional file 1. Supplementary Tables 1–3 and supplementary Figures 1–16. [file 40793_2024_547_MOESM1_ESM.docx]

**Supplementary Table 1**. Distribution of samples for shotgun sequencing.

| Sampling Location | Number of Beach Action Value Exceedance Day Samples | Number of Beach Action Value Non-Exceedance Day Samples |
| --- | --- | --- |
| Marie Curtis Park East Beach | 9 | 9 |
| Sunnyside Beach | 3 | 3 |
| Etobicoke Creek | 18 | |
| Humber River | 6 | |

**Supplementary Table 2**. Metagenomics and qPCR Quality Analytics.

| **Data Analytics** | **Number of Sequences (Mean ± Standard Deviation)** | **Sampling Locations** |
| --- | --- | --- |
| Before Quality Filtration | 12,925,145 ± 3,216,845 | Marie Curtis Park East Beach and Etobicoke Creek |
| After Quality Filtration | 10,694,704 ± 2,788,732 |  |
| Before Quality Filtration | 13,739,200 ± 3,523,743 | Sunnyside Beach and Humber River |
| After Quality Filtration | 11,479,959 ± 3,204,589 |  |
| **qPCR Standard Curve Quality Parameters** | | |
| **Quality Parameter** | **Value** | **Assay Type** |
| Calibration curve (R^2^) | 0.992 | *Enterococcus* (LsrRNA, 23S rRNA) |
| Slope | -3.25 |  |
| Intercept | 38.66 |  |
| Efficiency (%) | 100 |  |
| Calibration curve (R^2^) | 0.9998 | Microcystin (mcyE) |
| Slope | -3.37 |  |
| Intercept | 38.51 |  |
| Efficiency (%) | 97.84 |  |
| Calibration curve (R^2^) | 0.9996 | Saxitoxin (sxtA) |
| Slope | -3.33 |  |
| Intercept | 38.61 |  |
| Efficiency (%) | 99 |  |
| Calibration curve (R^2^) | 0.9996 | Cylindrospermopsin (cyrA) |
| Slope | -3.29 |  |
| Intercept | 38.92 |  |
| Efficiency (%) | 101 |  |
| Calibration curve (R^2^) | 0.9999 | Total Cyanobacteria (16S rRNA) |
| Slope | -3.46 |  |
| Intercept | 39.13 |  |
| Efficiency (%) | 94 |  |


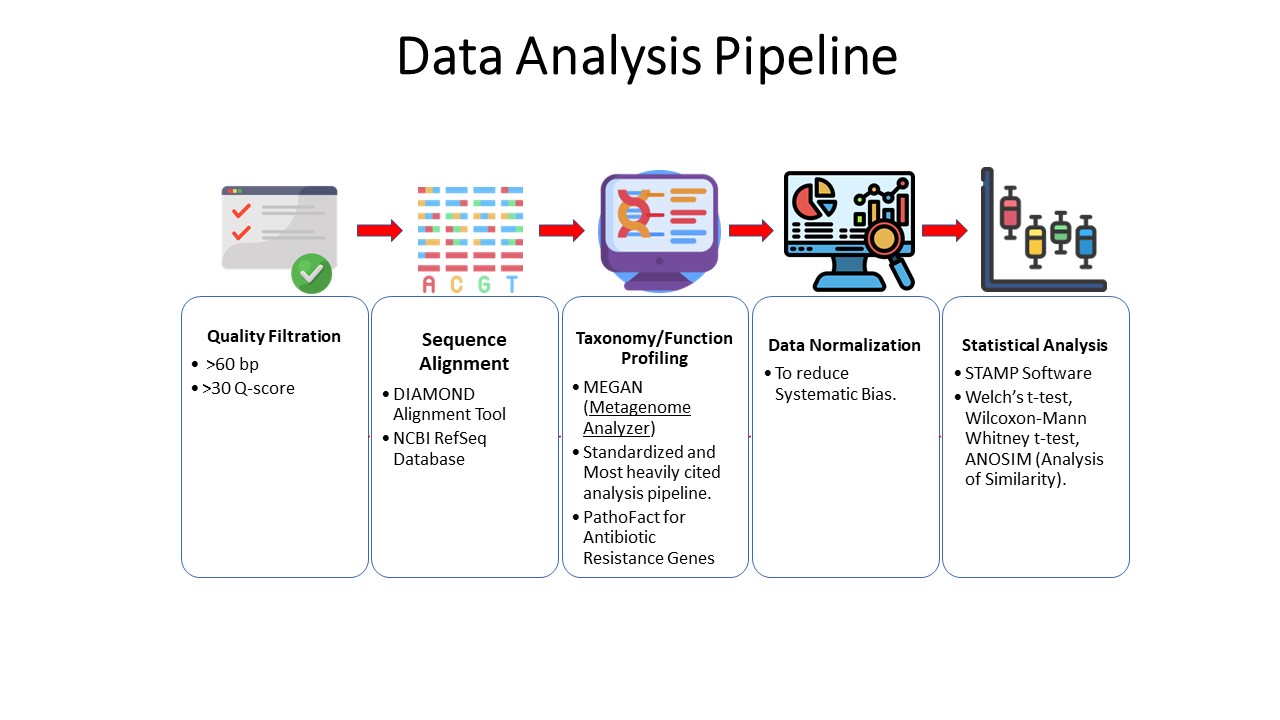
**Supplementary Figure 1.** Flowchart describing data filtration and analysis.


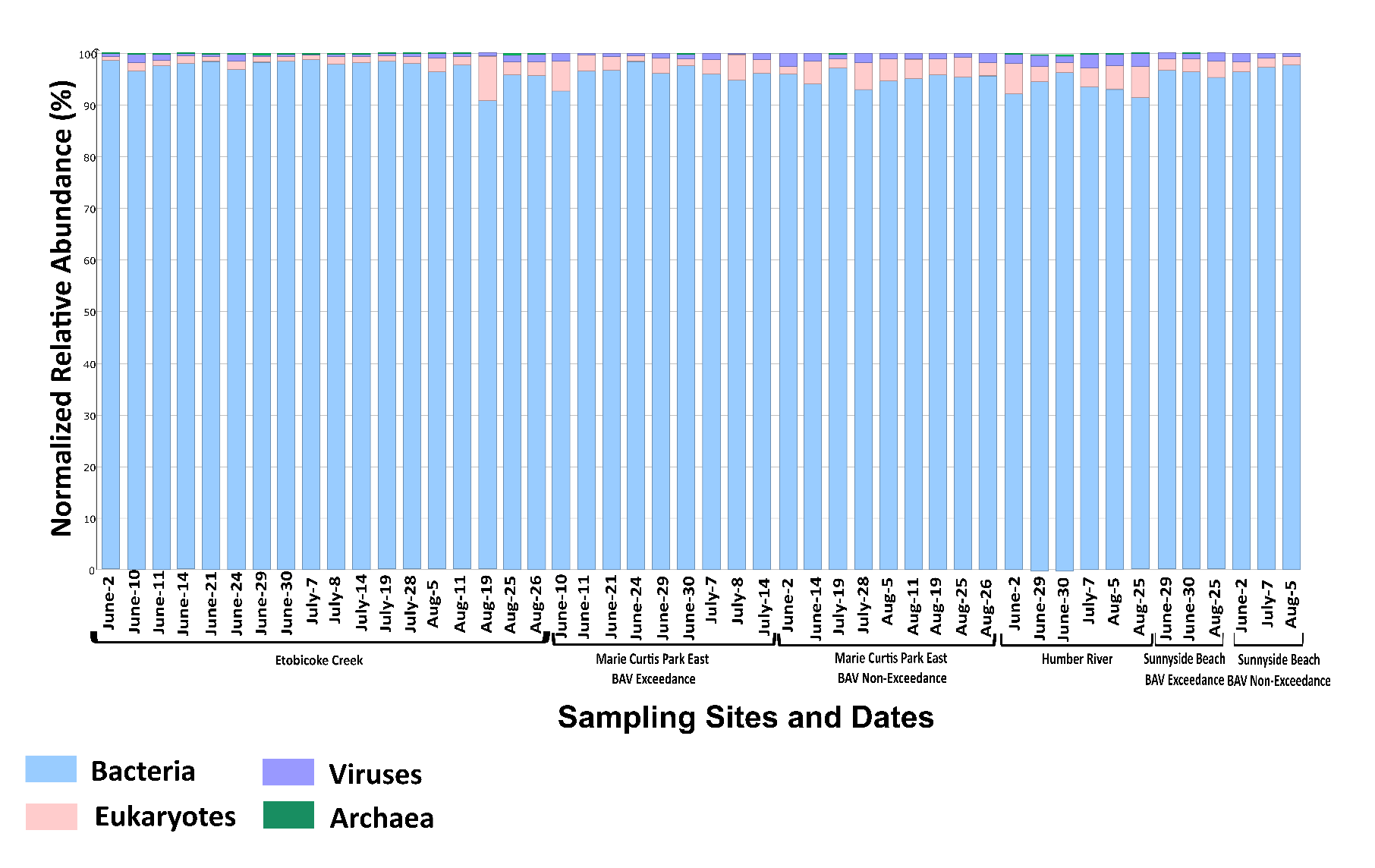


**Supplementary Figure 2.** Relative percentage taxonomic abundance of samples from Marie Curtis Park East Beach, Sunnyside Beach, Etobicoke Creek, and Humber River on Kingdom Level.


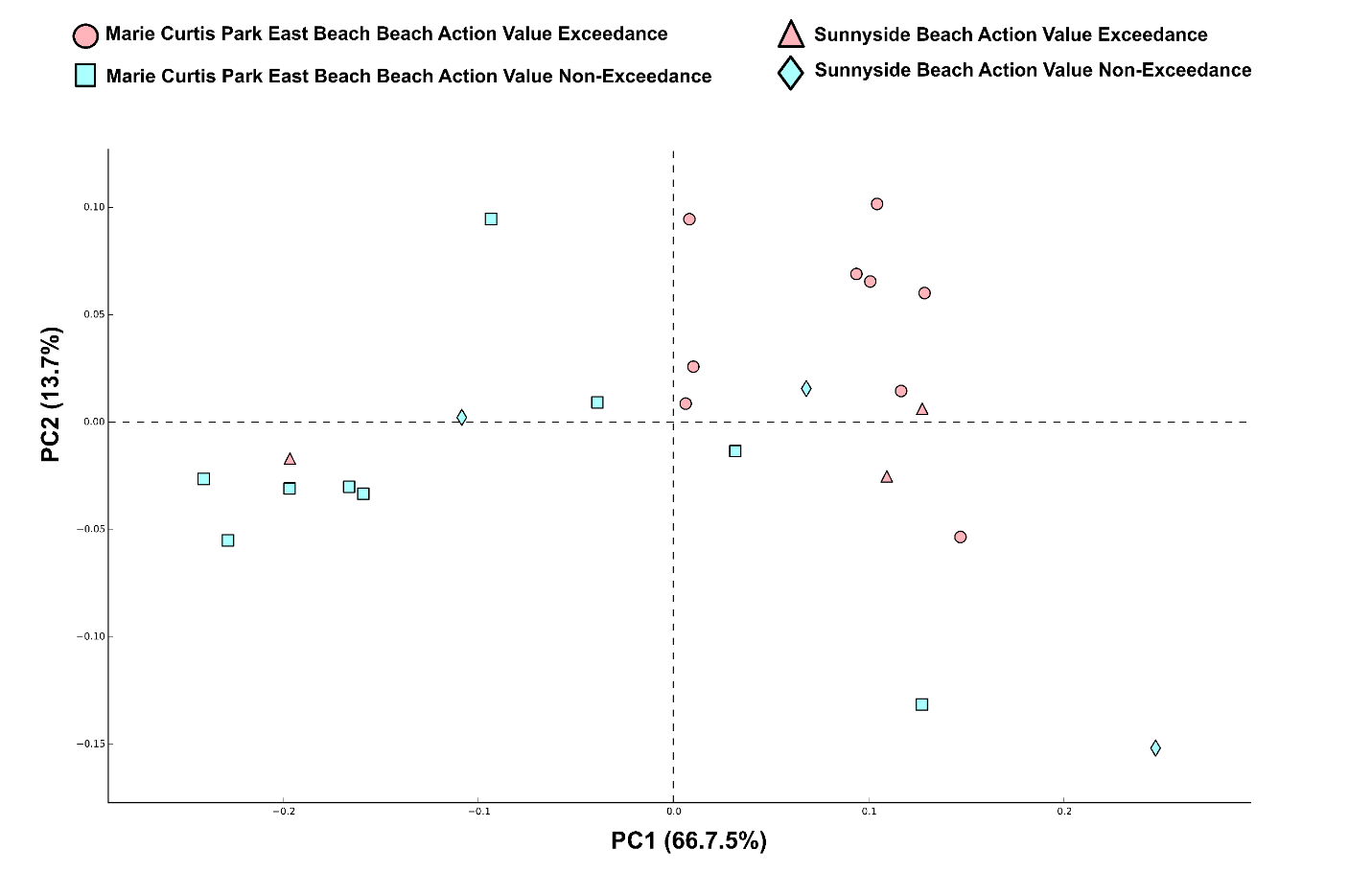


**Supplementary Figure 3.** Differential abundance Principal Component Analysis plots for Beach Action Value Exceedance and Non-Exceedance samples from Marie Curtis Park East and Sunnyside Beaches.


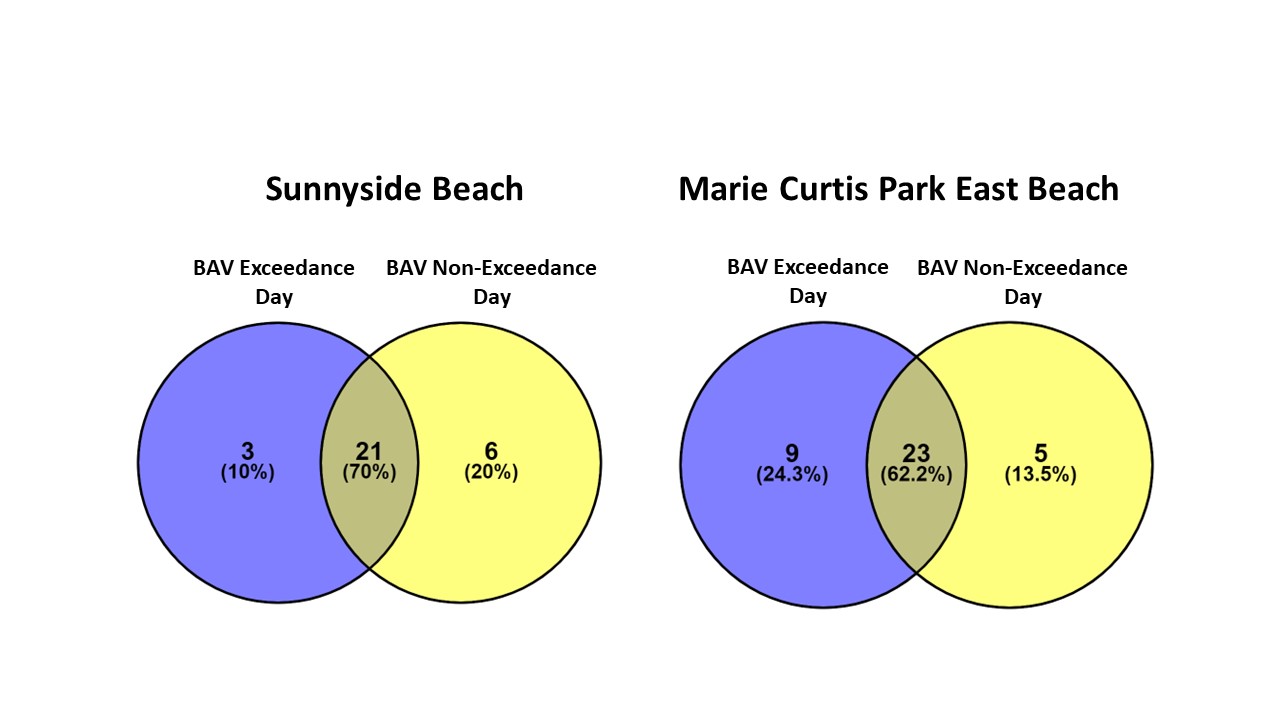


**Supplementary Figure 4.** Venn Diagram Analysis of Shared/Unique bacterial genera in Core Microbiome between Beach Action Value (BAV) Exceedance Beach Days and Non-Exceedance Beach Days from Marie Curtis Park East and Sunnyside Beaches.


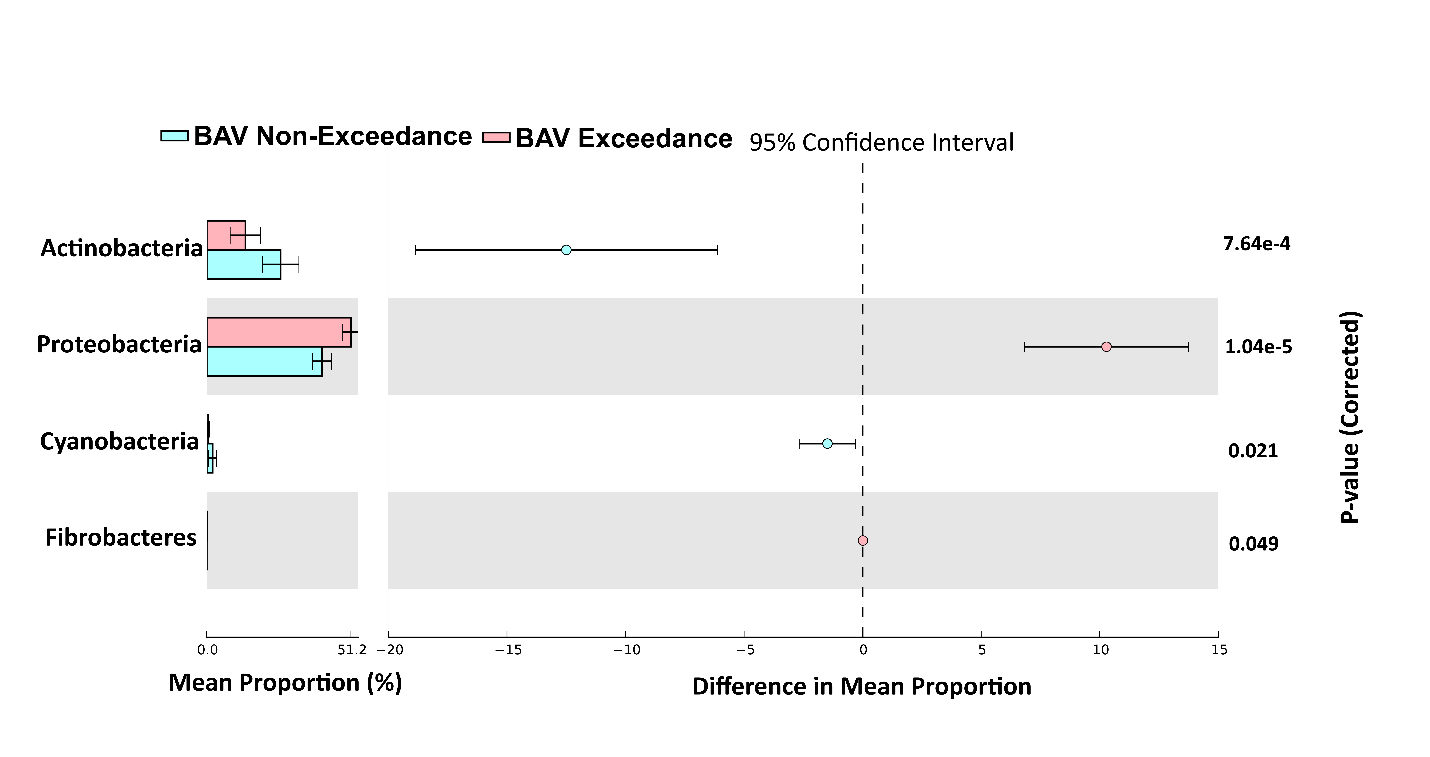


**Supplementary Figure 5.** Differential abundance extended error bar plot on phylum level for Beach Action Value Exceedance and Non-Exceedance samples from Marie Curtis Park East Beach.


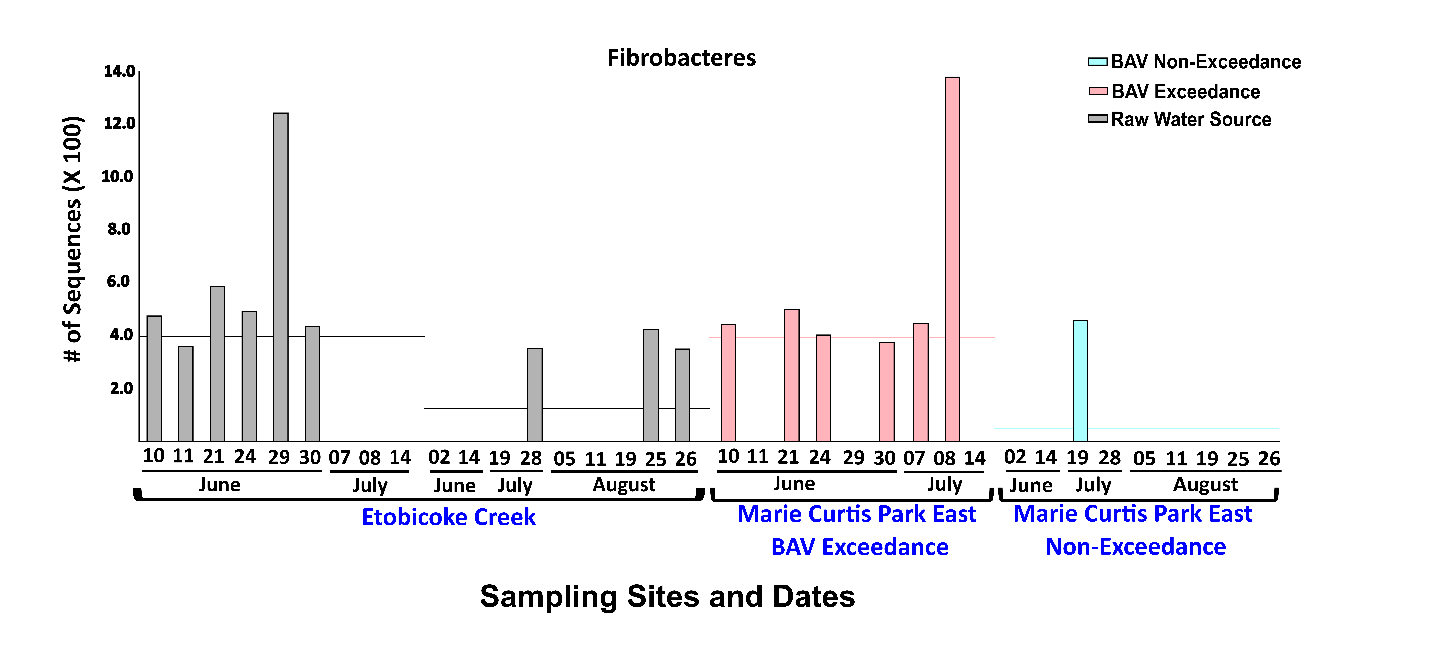


**Supplementary Figure 6.** Bar plot of Fibrobacteres abundance for Marie Curtis Park East Beach and Etobicoke Creek samples. The horizontal line represents the average number of normalized sequences for each group.


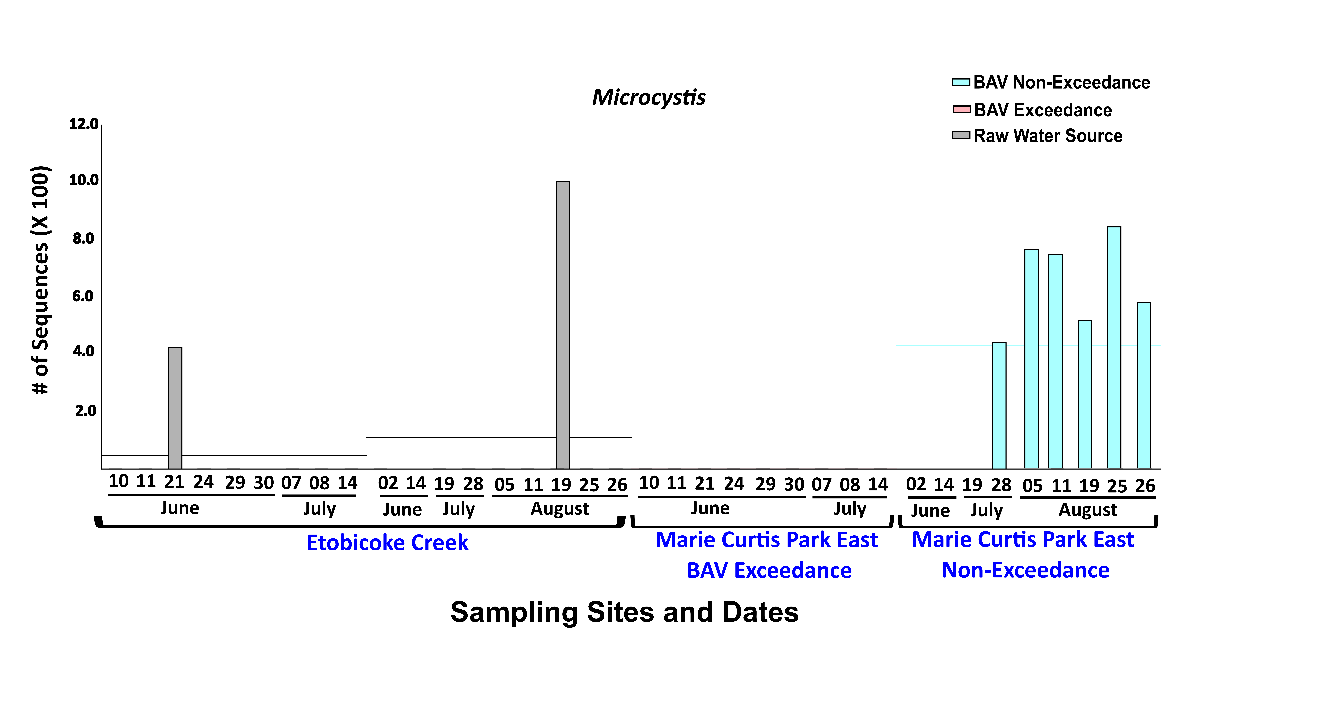


**Supplementary Figure 7.** Bar plot of *Microcystis* abundance for Marie Curtis Park East Beach and Etobicoke Creek samples. The horizontal line represents the average number of normalized sequences for each group.


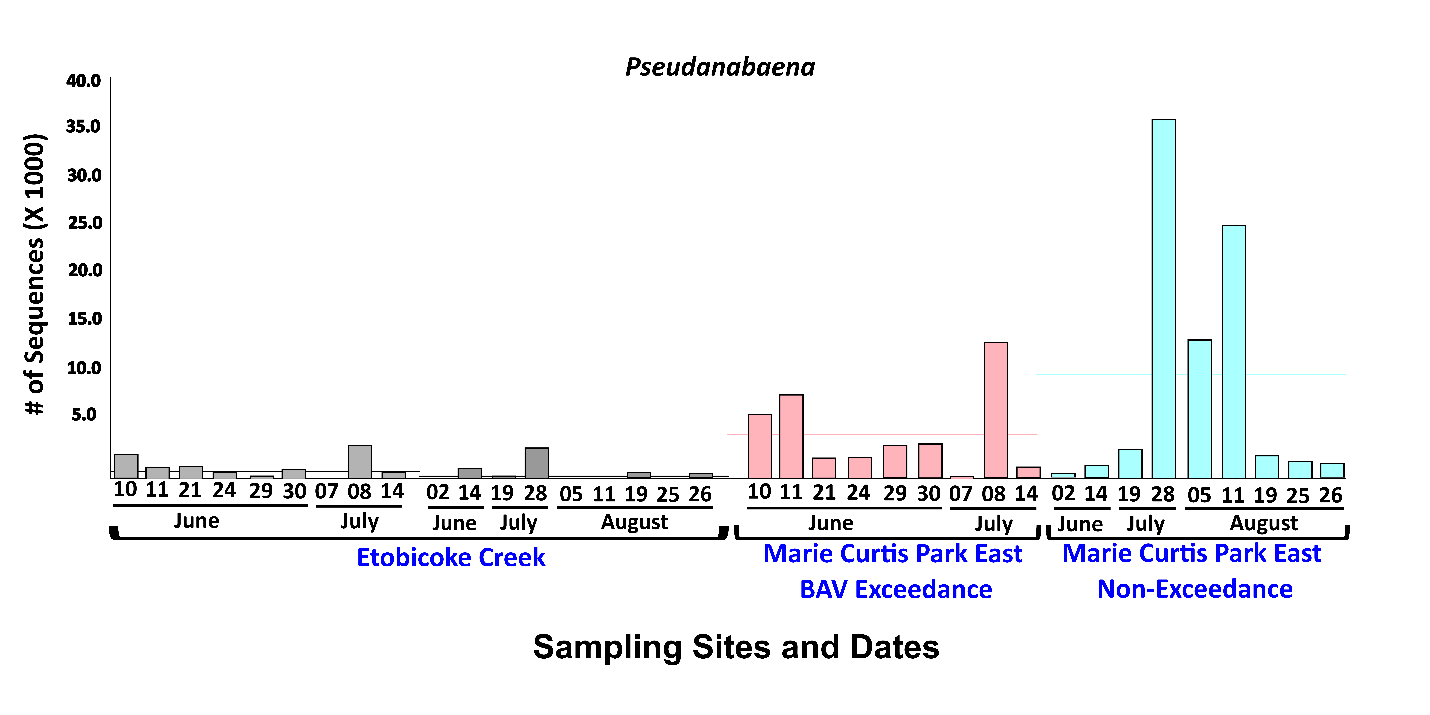


**Supplementary Figure 8.** Bar plot of *Pseudanabaena* abundance for Marie Curtis Park East Beach and Etobicoke Creek samples. The horizontal line represents the average number of normalized sequences for each group.


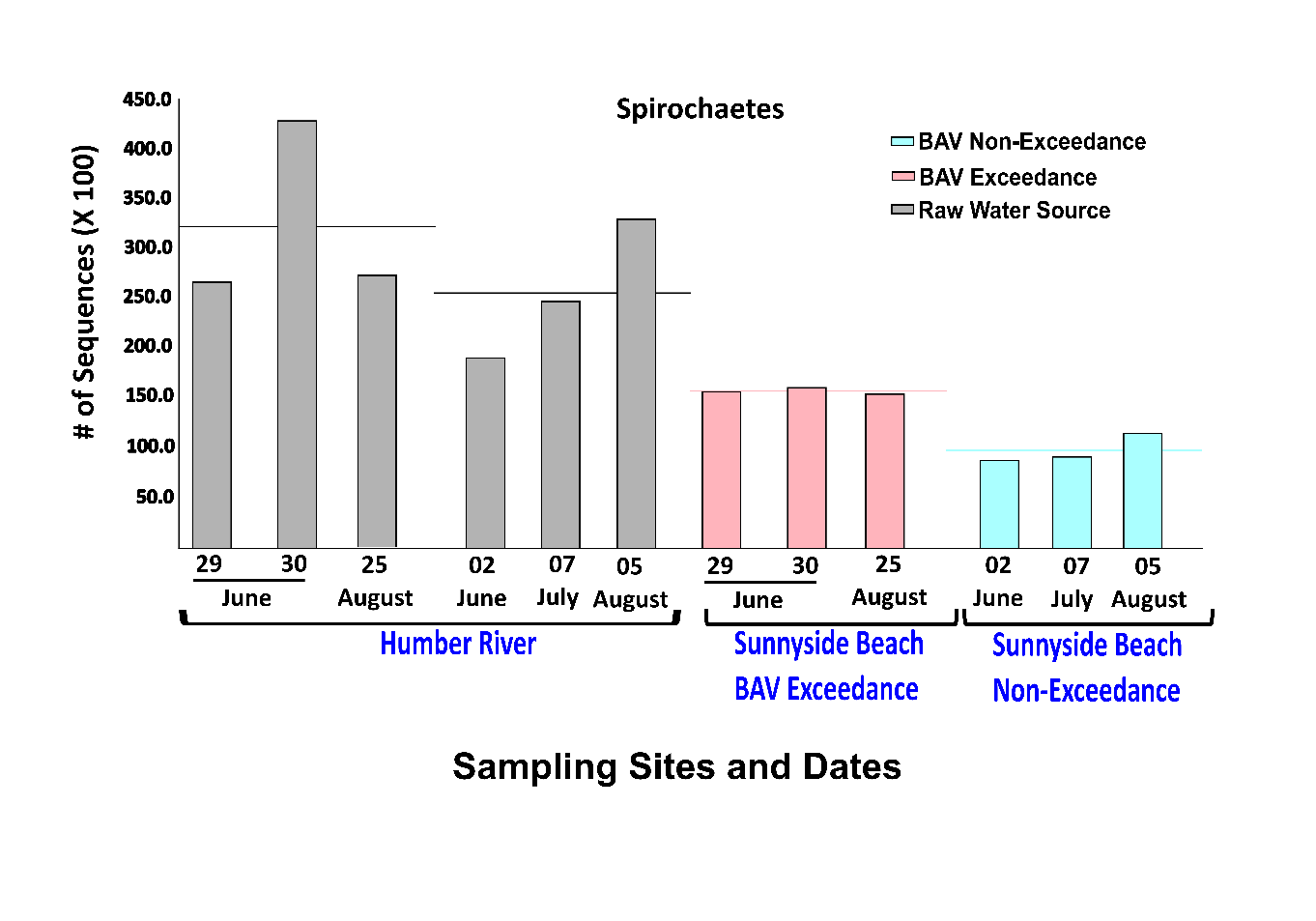


**Supplementary Figure 9.** Bar plot of Spirochaetes abundance for samples from Sunnyside Beach and Humber River. The horizontal line represents the average number of normalized sequences for each group.


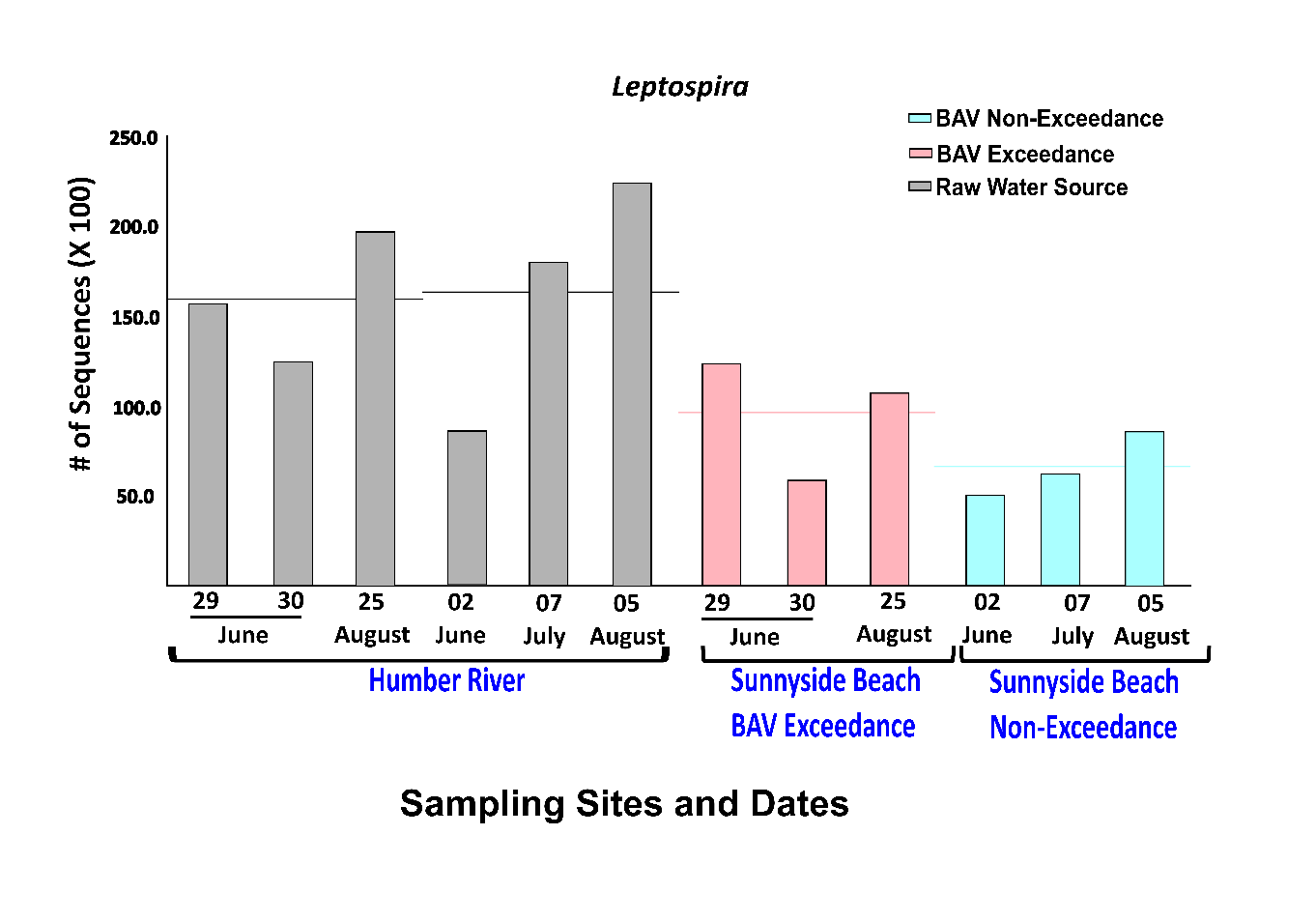


**Supplementary Figure 10.** Bar plot of *Leptospira* abundance for samples from Sunnyside Beach and Humber River. The horizontal line represents the average number of normalized sequences for each group.


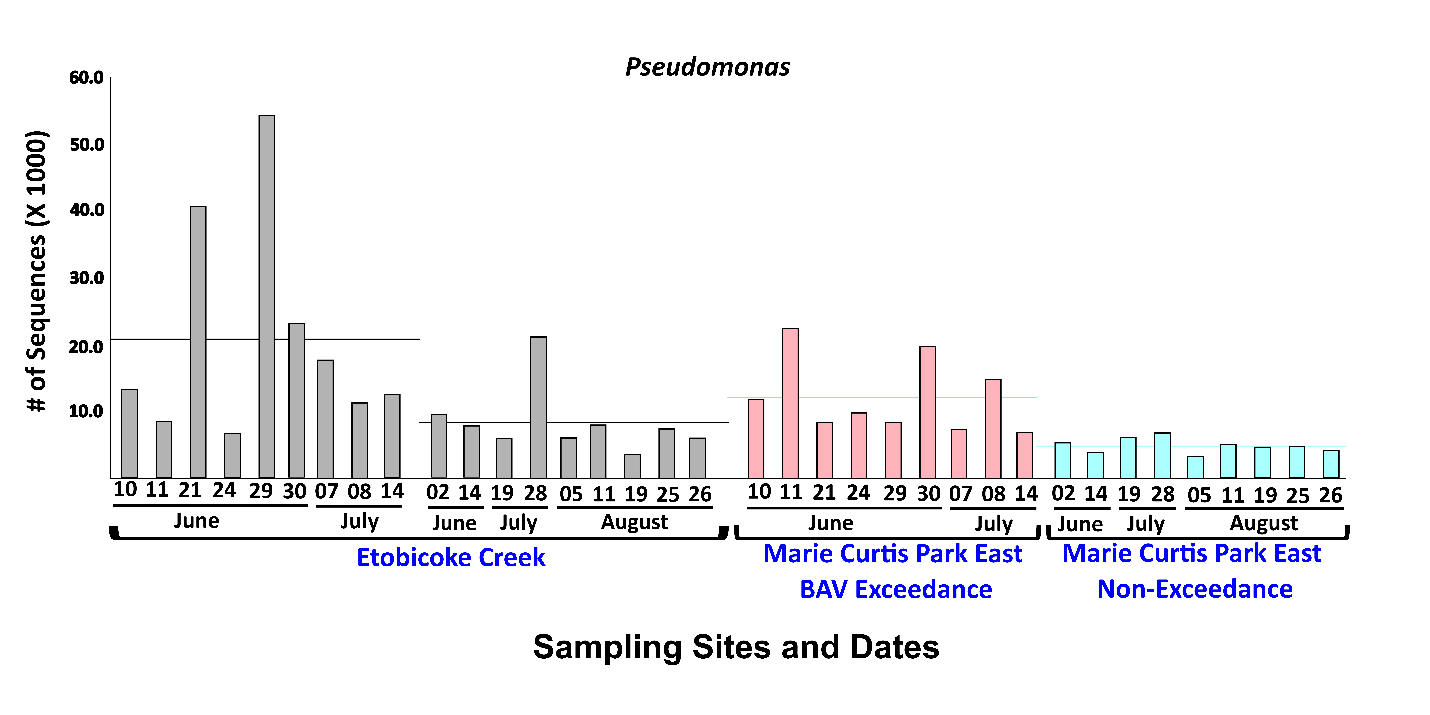


**Supplementary Figure 11.** Bar plot of *Pseudomonas* abundance for Marie Curtis Park East Beach and Etobicoke Creek samples. The horizontal line represents the average number of normalized sequences for each group.


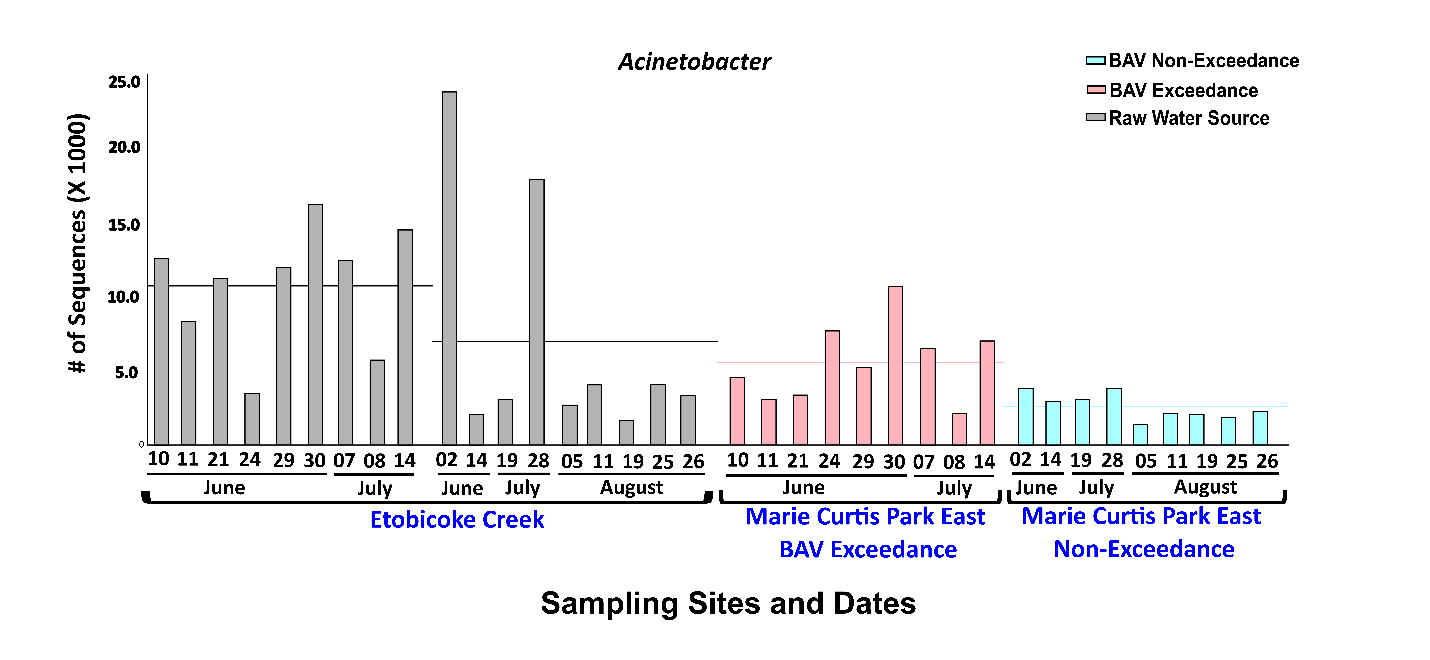


**Supplementary Figure 12.** Bar plot of *Acinetobacter* abundance for samples from Marie Curtis Park East Beach and Etobicoke Creek. The horizontal line represents the average number of normalized sequences for each group.


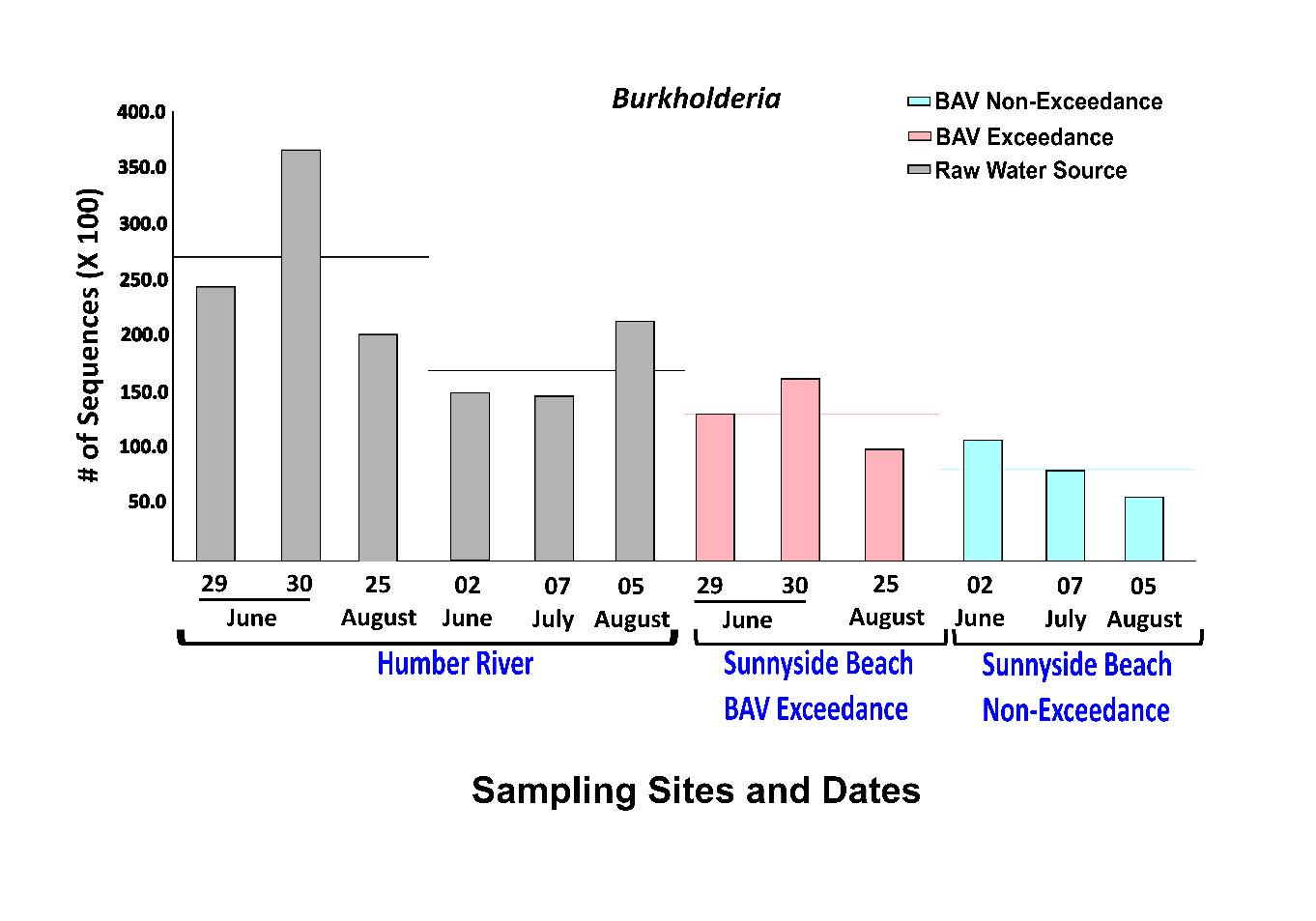


**Supplementary Figure 13.** The bar plot of *Burkholderia* abundance for Sunnyside Beach and Humber River samples. The horizontal line represents the average number of normalized sequences for each group.


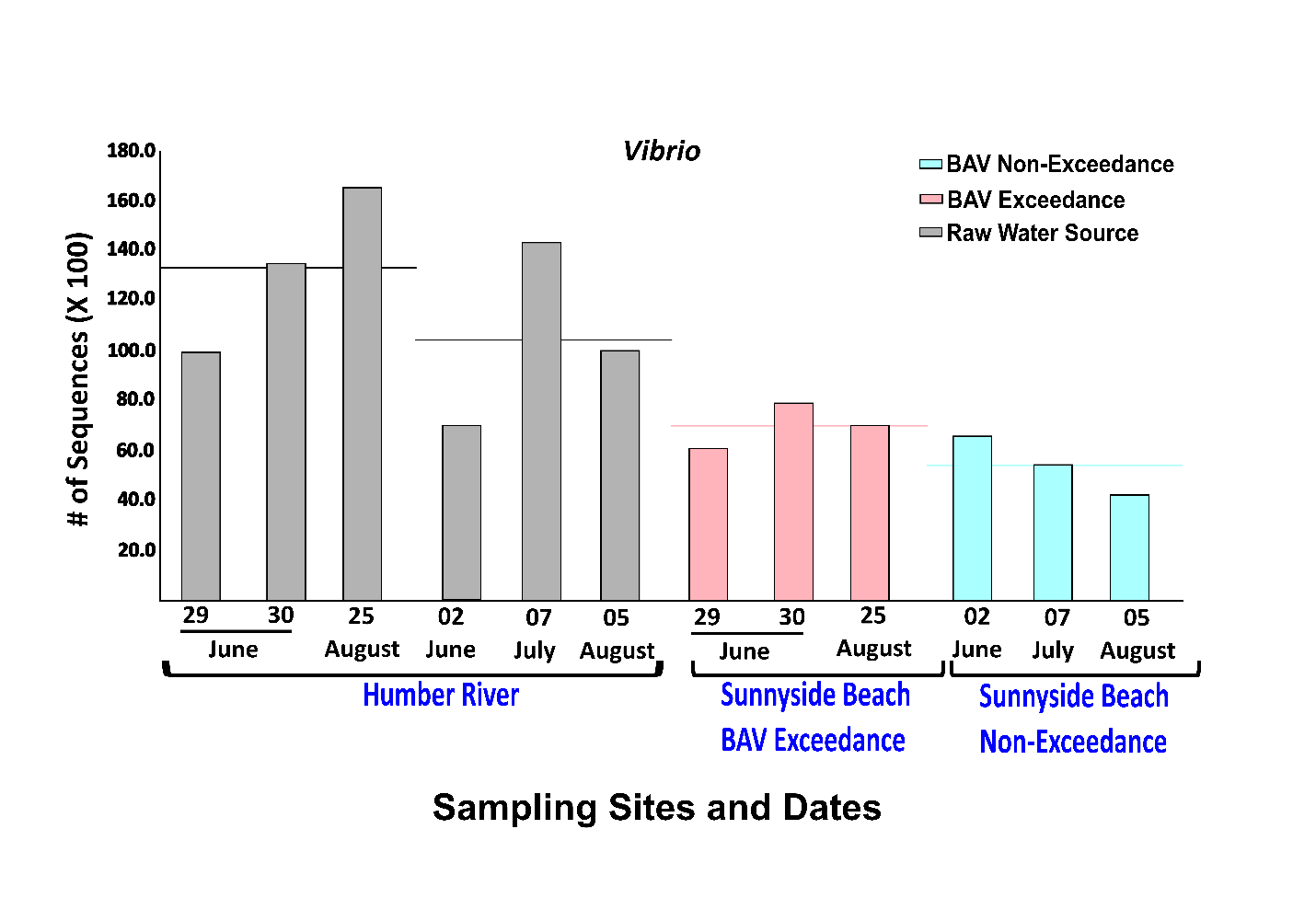


**Supplementary Figure 14.** Bar plot of *Vibrio* abundance for samples from Sunnyside Beach and Humber River. The horizontal line represents the average number of normalized sequences for each group.


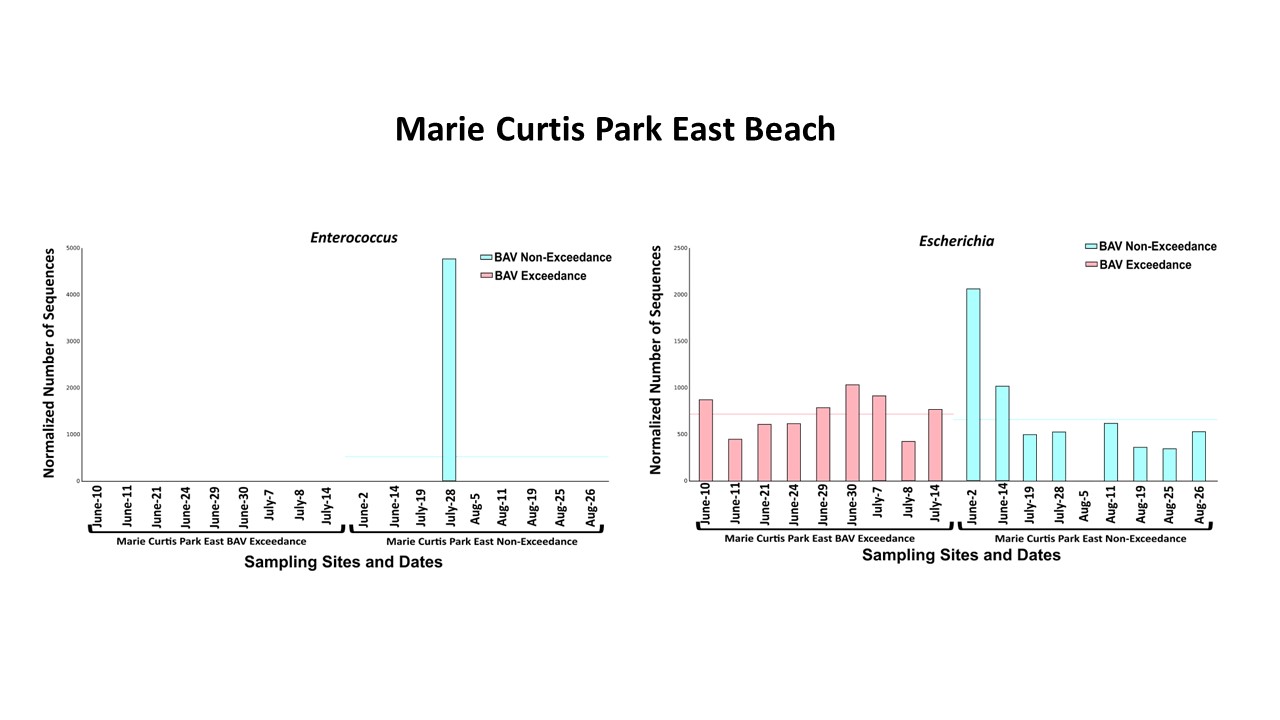


**Supplementary Figure 15.** Bar plots of *Escherichia* and *Enterococcus* abundance for Marie Curtis Park East Beach samples. The horizontal line represents the average number of normalized sequences for each group.


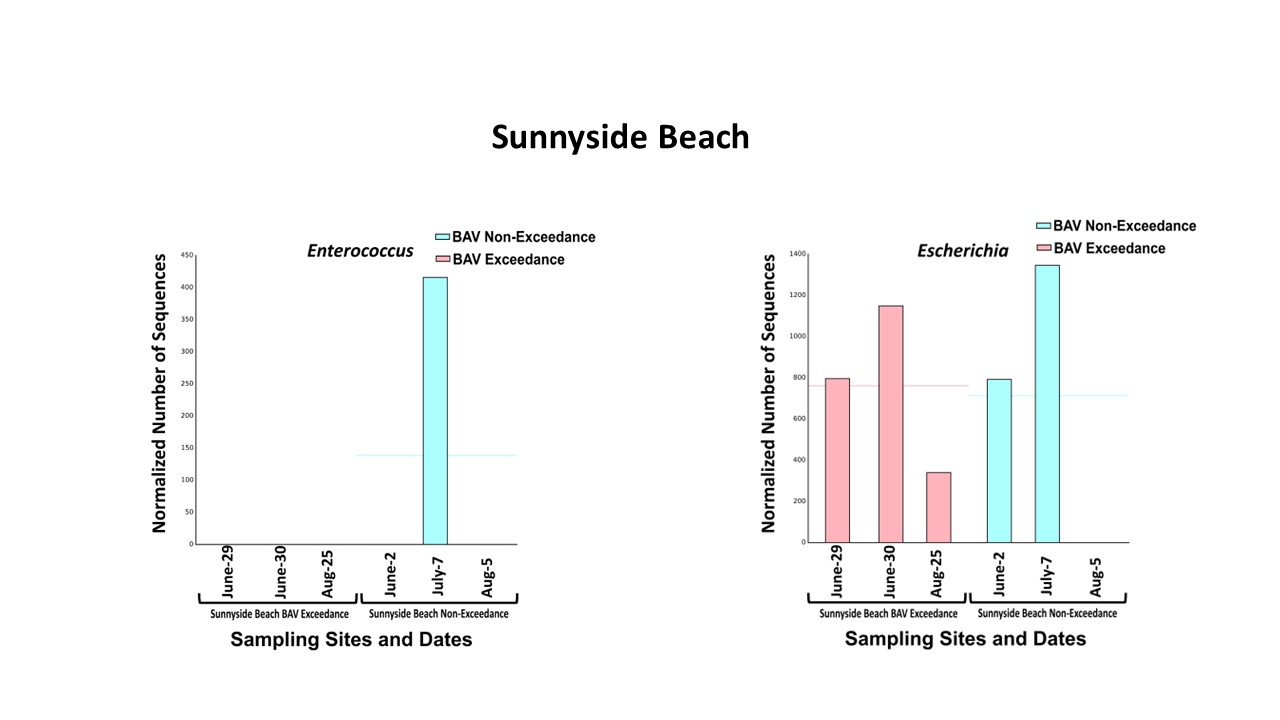


**Supplementary Figure 16.** Bar plots of *Escherichia* and *Enterococcus* abundance for samples from Sunnyside Beach. The horizontal line represents the average number of normalized sequences for each group.

**Supplementary Table 3.** Correlation analysis between Faecal indicator densities and Antibiotic Resistance Genes for Marie Curtis Park East Beach, Sunnyside Beach, Etobicoke Creek and Humber River.

| Faecal Indicator | Antibiotic Resistance | Correlation Coefficient | P-value | Sampling Site |
| --- | --- | --- | --- | --- |
| *E. coli* | Aminoglycoside Resistance | 0.4 | 0.09 | Marie Curtis Park East Beach |
|  | Beta-lactam Resistance | 0.1 | 0.4 |  |
|  | Multidrug Resistance | 0.2 | 0.3 |  |
|  | Tetracycline Resistance | 0.1 | 0.6 |  |
|  | Macrolides-Lincosamides Resistance | 0.09 | 0.7 |  |
| *Enterococcus* | Aminoglycoside Resistance | 0.3 | 0.2 | Marie Curtis Park East Beach |
|  | Beta-lactam Resistance | 0.08 | 0.7 |  |
|  | Multidrug Resistance | 0.1 | 0.4 |  |
|  | Tetracycline Resistance | 0.1 | 0.6 |  |
|  | Macrolides-Lincosamides Resistance | 0.03 | 0.8 |  |
| *E. coli* | Aminoglycoside Resistance | 0.2 | 0.2 | Etobicoke Creek |
|  | Beta-lactam Resistance | 0.1 | 0.5 |  |
|  | Multidrug Resistance | 0.1 | 0.6 |  |
|  | Tetracycline Resistance | 0.2 | 0.3 |  |
|  | Macrolides-Lincosamides Resistance | 0.3 | 0.1 |  |
| *Enterococcus* | Aminoglycoside Resistance | 0.3 | 0.1 | Etobicoke Creek |
|  | Beta-lactam Resistance | 0.2 | 0.2 |  |
|  | Multidrug Resistance | 0.1 | 0.6 |  |
|  | Tetracycline Resistance | 0.2 | 0.2 |  |
|  | Macrolides-Lincosamides Resistance | 0.3 | 0.1 |  |
| *E. coli* | Aminoglycoside Resistance | 0.02 | 1 | Sunnyside Beach |
|  | Beta-lactam Resistance | 0.3 | 0.5 |  |
|  | Multidrug Resistance | 0.02 | 1 |  |
|  | Tetracycline Resistance | 0.6 | 0.1 |  |
|  | Macrolides-Lincosamides Resistance | 0.05 | 0.9 |  |
| *Enterococcus* | Aminoglycoside Resistance | 0.4 | 0.4 | Sunnyside Beach |
|  | Beta-lactam Resistance | 0.5 | 0.2 |  |
|  | Multidrug Resistance | 0.5 | 0.2 |  |
|  | Tetracycline Resistance | 0.6 | 0.2 |  |
|  | Macrolides-Lincosamides Resistance | 0.6 | 0.1 |  |
| *E. coli* | Aminoglycoside Resistance | 0.4 | 0.3 | Humber River |
|  | Beta-lactam Resistance | 0.4 | 0.3 |  |
|  | Multidrug Resistance | 0.2 | 0.6 |  |
|  | Tetracycline Resistance | 0.5 | 0.2 |  |
|  | Macrolides-Lincosamides Resistance | 0.08 | 0.9 |  |
| *Enterococcus* | Aminoglycoside Resistance | 0.7 | 0.1 | Humber River |
|  | Beta-lactam Resistance | 0.8 | 0.06 |  |
|  | Multidrug Resistance | -0.8 | 0.03 |  |
|  | Tetracycline Resistance | -0.8 | 0.03 |  |
|  | Macrolides-Lincosamides Resistance | 0.4 | 0.3 |  |
